# Policy Standards for Healthy Built Environments: A Systematic Review of Health Impact Assessments and Nonrandomized Evaluations of Implemented Policies

**DOI:** 10.1007/s11524-026-01137-4

**Published:** 2026-09-19

**Authors:** Reint A. Huijzendveld, Famke J. M. Mölenberg, Frank J. van Lenthe, Mariëlle A. Beenackers

**Affiliations:** Department of Public Health, Erasmus MC, University Medical Center Rotterdam, Rotterdam, Netherlands

**Keywords:** Policy standards, Built environment, Health impact assessment, Natural experiment

## Abstract

The use of policy standards for the built environment is increasingly explored, yet evidence on their health impact is limited. This systematic review assesses the impact and projected effects of built environment policy standards on health, alongside factors influencing observed changes and lessons learned. We conducted a comprehensive search across 11 databases for studies published between 1990 and 2024 evaluating policy standards targeting the domains of publicly accessible urban green space (UGS), transportation, or food environment in upper-middle- and high-income countries and reporting a health-related outcomes. Prospective modeling and retrospective studies with before and after or comparison groups were eligible. Data extraction covered mortality, morbidity, health determinants, unanticipated findings and reported implementation lessons. Risk-of-bias assessment employed the ROBINS-I and PROBAST tools as well as an additional quality appraisal for HIAs. Of 11,247 records screened, 27 studies were included. Health Impact Assessments (HIAs) on UGS policy standards estimated, among other outcomes, up to 25 fewer projected deaths/100,000; one HIA on superblocks (mixed domains) projected twice that number. A HIA on a transport standard projected 3.1% less cardiovascular morbidity. In evaluations of applied policy standards, a transport intervention was associated with more walking. Two of three applied food stocking studies were associated with increased healthy food sales. In mixed-domain studies, the Liveable Neighborhood Guidelines were associated with increased odds for walking outcomes and sense of community, and studies on 20-minute neighborhoods were associated with increased odds for walking outcomes, but no change in BMI or food-related outcomes. Several applied studies reported unplanned effects such as spillover effects of the intervention to neighboring areas and increased revenues. HIAs highlighted addressing gentrification and ensuring quality of UGS. The wide range of outcomes and methods limited comparability across studies, while the modeling nature of HIAs and the observational design of most applied studies complicated the risk-of-bias assessment and generally resulted in low certainty of evidence. Many applied policy standards were associated with beneficial health behaviors, suggesting applied policy standards can be effective for improving health outcomes. HIAs projected health effects of various extents. However, it remains important to assess potential unintended consequences and evaluate whether policy standards contribute to reducing health inequalities.

**Supplementary Information:**

The online version contains supplementary material available at https://doi.org/10.1007/s11524-026-01137-4.

## Introduction

In 2050, almost 70% of the world’s population is projected to live in cities, highlighting the role of urban planning in public health [1]. The built environment plays a key role in shaping health and well-being, and socioecological models recognize environmental factors as major determinants of health [2]. Accordingly, the World Health Organization (WHO) identifies the creation of healthy environments as a cornerstone of primary prevention [3]. Key features of healthy built environments include accessible urban green space (UGS), healthy food environment, and active transportation infrastructure [4, 5]. However, access to these resources is unequally distributed [6], with lower-income populations experiencing reduced access to UGS [7], healthy food [8], and active transport infrastructure [9]. Well-planned urban environments can reduce behavioral risk factors and disease burden, whereas poorly planned environments are associated with higher morbidity and mortality [4, 5].

UGS, food environments, and transportation systems influence health through different pathways. UGS may improve health through mitigation (reducing exposure to environmental stressors), restoring capacities (improving attention and reducing stress), and building capacities (promoting physical activity and fostering social cohesion) [10]. Food environments shape dietary behavior [11], yet current food environments encourage unhealthy food choices. Prevention efforts tend to focus on individualized educational interventions, while a population-level approach involves modifying the food environment by improving access to healthy foods while limiting unhealthy options [12]. Studies associated fast-food outlet proximity and presence with increased obesity risk, whereas proximity to supermarkets and higher density of fruit and vegetable outlets were linked to reduced obesity risk [13, 14]. Similarly, active transport infrastructure and public transport accessibility can promote physical activity [15] while reducing exposure to air pollution and noise [16]. Together, improvements across these domains may reduce the burden of chronic diseases and mortality [16–19]. However, evidence remains limited regarding the most effective and sustainable approaches for implementing healthier built environments.

One potential approach is the implementation of built environment policy standards (hereafter referred to as policy standards). Similar to standards for environmental noise or air pollution, these standards specify measurable planning targets, such as minimum UGS coverage, restrictions on fast-food outlet proximity, or maximum walking distances to public transport. When effectively communicated, they provide a practical framework for supporting healthier urban planning and can promote a more equal resource distribution [6]. The use of policy standards is increasingly explored [20], but has limitations Their health effects have rarely been evaluated in real-world settings, many standards emphasize quantity rather than quality, and little is known about unintended consequences [6, 20]. Therefore, it remains unclear whether policy standards can serve as effective tools for improving public health.

This is not the first review of built environment policy standards. Mueller et al. (2021) developed practical urban and transport planning principles through a narrative review and stakeholder workshop [20], identifying four priorities for healthier cities: fostering compact cities, decreasing dependence on private motorized vehicles, active transport, and expanding green and public space. Their approach was conceptual and policy-oriented and aimed at developing guiding principles. We extend this evidence by conducting a systematic review of empirical studies evaluating both implemented and modeled policy standards, while additionally including food environment policies. This systematic review aims to assess the impact and projected effects of applied and modeled policy standards on health and environmental outcomes and to identify factors that explain observed changes.

## Methods

The protocol of this study was registered in April 2023 at PROSPERO (CRD42023414767). Our systematic literature search followed the Preferred Reporting Items for Systematic Reviews and Meta-Analyses (PRISMA) guidelines [21].

### Search Strategy

Eleven scientific databases (Medline, Embase, Web of Science, Psycinfo, Cochrane Central Register of Controlled Trials, IBSS, Social Science Database, ASSIA, Social Services Abstracts, Sociological Abstracts, Sociology Database) were searched for articles evaluating policy standards. The search terms were constructed from four parts, including (1) synonyms for public policies, standards, and concepts; (2) synonyms for built, green, food, and transport environments; (3) synonyms for healthy behaviors, health-related social factors, and health outcomes; and (4) a term excluding animal or nonhuman studies, conference abstracts, book chapters, and dissertation abstracts. The detailed search terms are listed in Supplementary Table 4. To identify additional relevant studies, we hand-searched the reference lists and citations of the studies included. Publication dates were limited to studies published between 1990 and December 2024. The language requirement was having an English abstract.

### Study Selection and Eligibility Criteria

**Fig. 1:**
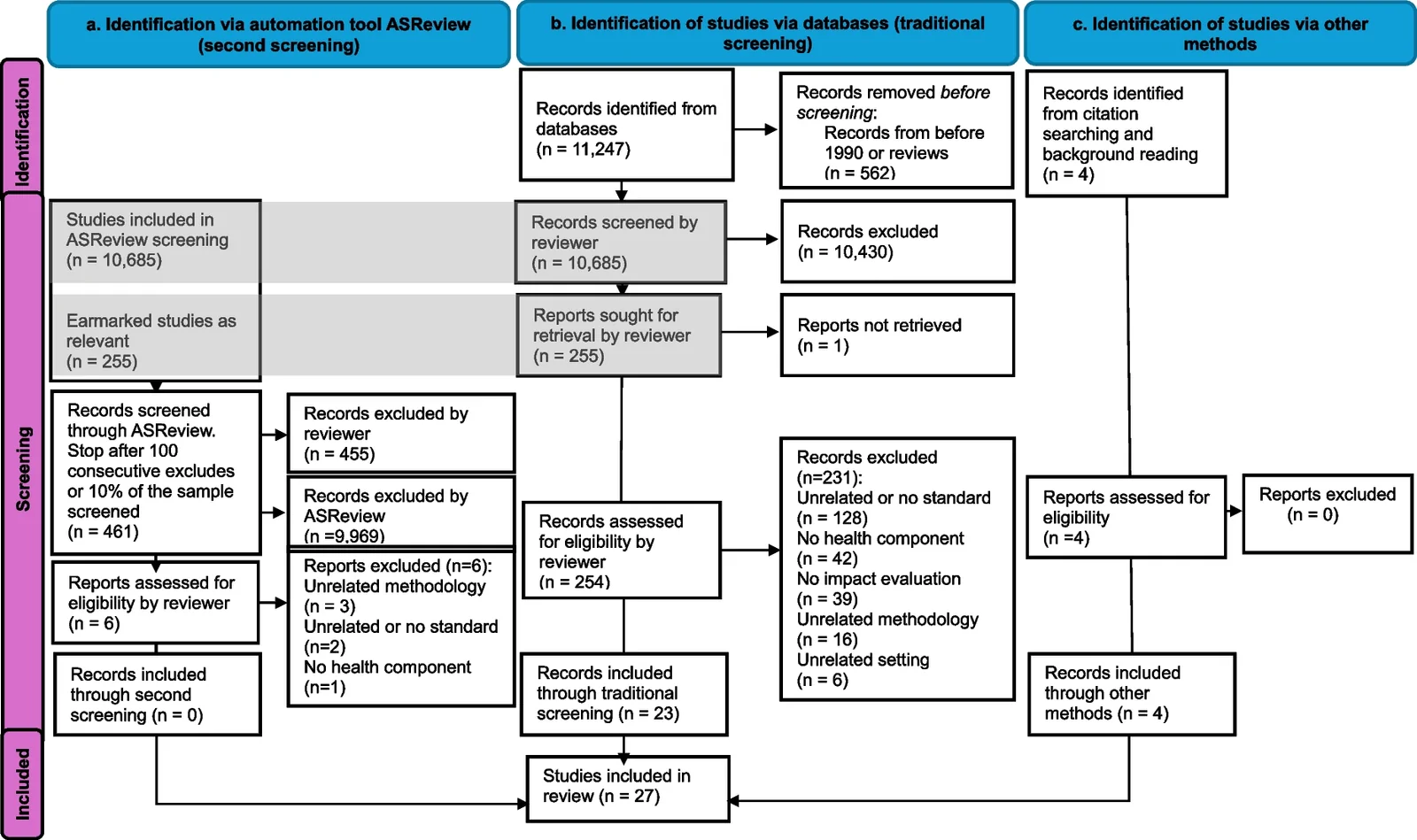
Flow diagram of study inclusion. **a** Second screening via ASReview. **b** Traditional screening, all records were manually reviewed. **c** Other methods

The selection process was a two-step approach: first, one reviewer (RH) hand-screened all articles via EndNote, using the traditional screening method to ensure all records were seen and assessed by a reviewer (RH). After manual title-abstract screening, full-text records were retrieved and reviewed, and all potentially included articles were discussed with the team. A second reviewer (MB) performed an additional screening with ASReview [22] to ensure no relevant articles were excluded. ASReview uses multiple machine learning models to help researchers select articles more efficiently by incorporating their inclusion decisions and ranking studies by relevance. The included studies from manual title-abstract screening were earmarked, and along with all other records fed into ASReview. Here, we employed a data-driven stopping heuristic of either 100 consecutive irrelevant records or reviewing 10% of all records, whichever came first. Relevant records were screened similarly to manual full-text screening. According to the SAFE procedure, this second screening aligns with the quality check phase, which aims to detect any relevant records that may have been wrongly excluded [23]. Our review strategy was designed to ensure robust algorithm performance, allowing us to screen only a subset of records in the second phase, which was deemed sufficient to meet our objectives. See Fig. 1 for specifications on the number of records screened and included.

Studies that evaluated policy standards and their effects on health determinants or health outcomes, either prospectively or retrospectively, were included. Prospective evaluations comprised Health Impact Assessments (HIAs), defined as quantitative modelling studies that projected the potential health impacts of theoretical policy standards. Retrospective evaluations comprised implemented policy evaluations, defined as empirical studies that assessed the observed health impacts of real-world, implemented policy standards using observed population data rather than modelled projections. Policy standards could be part of a broader policy package that included elements without policy standards. Examples of policy standards include a minimum amount of UGS/capita, a maximum number of unhealthy food outlets/neighborhood, or target proportions of bicycle lanes in a road network. It was essential that these policy standards had a denominator and were expressed as absolute values (e.g., the target value for UGS/person is *x* m^2^, where *x* can be an absolute value or a percentage) rather than relative changes from the current situation (e.g., the target value for UGS/person is increased by *x* m^2^). These relative changes are not transferable to different settings and can be repeated indefinitely. We did not focus on specific terminology for standards; terms such as norms, thresholds, goals, or guidelines were all included.

The eligibility criteria were that policy standards were:

1. aimed at changes in the built environment within the three domains of UGS, food environment, or transport environment,
2. intended to be applied by local, regional, or (inter)national governmental bodies,
3. related to publicly accessible places, such as streets, parks, food stores, and outlets (excluding schools, paid sports facilities, or workplaces),
4. affected the built environment on a neighborhood or larger scale,
  Furthermore, studies had to:
5. report at least one health determinant or health-related outcome,
6. include before-after designs, cross-sectional designs with at least one comparison group, or prospective modeling scenarios,
7. include populations located in high- and upper-middle-income countries, according to the World Bank country classification [24].

Studies were excluded if they:

1. compared attributes of the built environment between locations, without evaluating policy standards,
2. assessed policy-related thresholds that do not affect the built environment, such as recommended daily intakes,
3. assessed policy standards for behavioral consequences of changes in the built environment, such as targets for the proportion of the population that cycles,
4. assessed policies aimed at increasing amenities without a denominator, such as “one million more trees.”

### Data Extraction

Data from the included studies were extracted by one researcher (RH), and 20% of the sample was verified by other researchers (MB and FM). A custom-made standardized data extraction form was used, including details about the policy standard (theme, description, rationale, legal framework, timing, setting/country), study design (data source, recruitment, population size and characteristics, analysis, control variables), outcomes (main, additional, and unplanned outcomes), guidance on future implementation (lessons learned and policy recommendations), and the risk-of-bias assessment. Given the wide range of reported outcomes, we focused on extracting the main or most relevant health outcomes, as identified by the authors of the included study or our research team. We prioritized data from maximally adjusted models with minimal stratification. If reported, we extracted changes in the targeted environment as well as differential effects for socioeconomic, racial, ethnic, or migration groups. EndNote was used as a database for references. When studies met the eligibility criteria but relevant key information was missing, the authors were contacted to provide further information.

For the applied studies, we combined two bias risk assessment tools due to the diversity in study designs and outcomes: Prediction model Risk Of Bias ASsessment Tool (PROBAST) [25, 26] for participant selection and the Risk Of Bias In Non-Randomized Studies - of Interventions (ROBINS-I) tool [27] for all other bias categories. The ROBINS-I tool indicated no selection bias risk inherent to the study designs included in this review. Thus, we utilized PROBAST to evaluate how participant exclusions were managed, ensuring criteria were clearly defined, justified by literature or logic, and relevant to the research question. We assessed biases related to confounding, participant selection, intervention classification, deviations from intended interventions, missing data, outcome measurement, and reported result selection.

For HIAs, the abovementioned tools did not adequately capture methodological variation, so we conducted a separate quality appraisal using a purpose-built set of criteria developed for this review. The appraisal assessed: (1) the quality and appropriateness of exposure–response functions (ERFs), (2) the plausibility of exposure scenarios and assumptions, (3) treatment of uncertainty and sensitivity analyses, (4) transferability of ERFs to the target population and setting, and (5) transparency and reproducibility. Domains were assessed via predefined, coarse criteria, with higher scores indicating greater methodological robustness (see Supplementary Table 5). This appraisal was not based on a validated instrument; therefore, results are descriptive and should be interpreted with caution.

Certainty in the body of evidence was assessed via the Grading of Recommendations Assessment, Development, and Evaluation (GRADE) guidelines [28]. This assessment was performed separately per outcome category across the body of evidence (see Supplementary Table 6). Because all included studies were non-randomized, certainty initially started at low. Evidence was then evaluated for risk of bias, inconsistency, indirectness, and imprecision. Publication bias was not formally assessed due to the small number and heterogeneity of studies per outcome.

### Data Synthesis

In a narrative synthesis, we described how policy standards related to health determinants and outcomes, and if possible, harmonized outcomes were reported for better comparability across studies. When reported in the study, factors contributing to changes, or unplanned changes in exposure or outcomes, were described. Studies were organized by design (retrospectively assessed applied policy standards, or prospectively through HIAs) and by standards theme. The quality of a HIA depends on the quality and assumptions of the underlying evidence, and it projects what could occur under ideal implementation. Therefore, they should be interpreted as what would happen in theory, rather than as empirical evidence of real-world policy effectiveness.

### Deviations from Prospero

In the protocol, a thematic content analysis was proposed to be used as analysis method. Instead, we analyzed the data with a narrative synthesis because this appeared to fit better with the type of studies collected. Additionally, the strategy for the second screening with ASReview was changed after reviewing various strategies [23]. Originally, we proposed a traditional approach of manually double screening of abstracts and discussing our inter-rater agreement. Instead, we used a traditional approach for the first reviewer and then extensively discussed the included studies of the first traditional screening. For the second reviewer, we added all included abstracts to the algorithm in ASReview and only checked for abstracts that may have been overlooked in the first screening.

## Results

### Study Characteristics

Of 11,247 records screened, 255 full-text articles were assessed, resulting in 27 included studies (Fig. 1) [29–55]. Sixteen studies were prospective HIAs [29–43], and twelve were nonrandomized evaluations of applied policy standards [44–55] (Table 1). Thirteen HIAs assessed UGS policy standards [29–41], three applied studies evaluated the food environment [52–54], one HIA [43] and one applied study [55] evaluated standards in the transport environment, and one HIA [42] and eight applied studies assessed mixed-domain standards [44–51]. All UGS studies were HIAs, predominantly conducted in Europe (two in the USA). Food environment studies, all from the USA, evaluated applied policy standards for minimum stocking requirements in stores, two as natural experiment using a controlled before-after designs [52, 53], and one as a case study without statistical testing [54]. Transport studies evaluated applied urban plans in the USA as a natural experiment using a cross-sectional design [55] and projected plans in a HIA in China [43]. Mixed-domain studies mainly assessed Australian applied urban plans, including three on livable neighborhood guidelines (LNG) (cross-sectional natural experiment) [49–51], five on 20-minute neighborhoods (20MNs) (cross-sectional observational study) [44–48], and one HIA on Superblocks in Spain [42]. Supplementary Figs. 2 and 3 show the geographical distribution and the harvest plot of outcomes, respectively.

**Table 1:** Study characteristics of the included studies and descriptions of the policy standards

Author	Standard theme (Study design)	Location	Study population (sample size)	Policy standard
Mueller (2017–1) [34]	UGS (Health Impact Assessment (HIA))	Barcelona, Spain	≥ 20 years old, Barcelona residents *N* = 1,357,361 (2012)	WHO universal access to urban green space (UGS): living within 300 m linear distance of urban green space (UGS) ≥ 0.5 ha. Operationalized in percentage green area (%GA) or normalized difference vegetation index (NDVI)
Mueller (2017–2) [33]	UGS (HIA)	Barcelona, Spain	≥ 20 years old, Barcelona residents *N* = 1,357,361 (2012)	
Mueller (2018) [32]	UGS (HIA)	Bradford, United Kingdom	Bradford residents having children 2007–2010 and ≥ 18 years old: *n* = 393,091 (2016) (*N* = 13,500)	
Nguyen (2024) [36]	UGS (HIA)	Barcelona, Spain	Barcelona children aged 2–17 years (*N* = 218,976)	
Barboza (2021) [35]	UGS (HIA)	978 cities, 49 greater cities in Europe	≥ 20 years old, residing in one of the cities in 2015 (*N* = 169,134,322)	
Iungman (2021) [29]	UGS (HIA)	Barcelona and Madrid, Spain	≥ 20 years old Barcelona residents in 2018 *N* = 1,348,800 ≥ 20 years old Madrid residents in 2018 *N* = 2,649,734	
Khomenko (2020) [31]	UGS (HIA)	Vienna, Austria	≥ 20 years old, Vienna residents 2017 (*N* = 1,488,832)	WHO universal access to UGS; alternative WHO policy standard: 9 m^2^/inhabitant of UGS, operationalized as %GA; Viennese policy: 3.5m^2^/inhabitant of UGS, operationalized as %GA. Taken up in development and master plans, but mandatory incentives are unclear
Dean (2024) [39]	UGS (HIA)	Denver and Phoenix, USA	Denver population ≥ 18: (*N* = 521,000), ≥ 35: (*N* = 298,000), ≥ 55: (*N* = 104,000) Phoenix population ≥ 18: (*N* = 1,110,000), ≥ 35: (*N* = 689,000), ≥ 65: (*N* = 252,000)	Tree canopy cover (TCC) goals: Denver’s Game Plan for a Healthy City: 20% TCC in each neighborhood. Halfway scenario: 16.5% TCC Phoenix’s Tree and Shade Master Plan: 25% citywide TCC. Halfway scenario: 13% TTC
Kondo (2020) [30]	UGS (HIA)	Philadelphia, USA	≥ 18 years old, Philadelphia residents 2014 (*N* = 1,600,000)	TCC goals: 30% citywide TCC. Taken up in development plans but mandatory incentives are unclear
Iungman (2023) [40]	UGS (HIA)	93 European cities	≥ 20 years old, residing in one of the 93 cities. (*N* = 57,896,852)	TCC goals: 30% per 250 × 250 m grid cell, as well as sensitivity analyses with 25% and 40% TCC
Mueller (2020) [42]	Mix: UGS/transport (HIA)	Barcelona, Spain	≥ 20 years old, Barcelona residents (*N* = 1,301,827 (2017)	Superblocks: In this concept, grid structured neighborhoods (1 × 1 blocks) are transformed to 3 × 3 superblocks with all inner roads having a calm interior for active transport and residential traffic at 20 km/h. Surrounding them is a basic road network for city-wide connectivity, allowing speeds up to 50 km/h. This network includes segregated cycling, pedestrian lanes, and bus lanes, with bus stops at major superblock intersections every 400 m
Vidal-Yañez (2023) [38]	UGS (HIA)	Barcelona, Spain	≥ 20 years old, residing in Barcelona in 2016 and 2017 (*N* = 3145)	Green Axis plan (Eixos Verds): The Eixos Verds plan transforms one-third of the streets into green corridors. The current pedestrian network occupies 16% of public space, implementing the Eixos Verds plan could raise this figure to 67%
Opbroek (2024) [37]	UGS (HIA)	Barcelona, Spain	Children aged 7–10-year-old, residing in Barcelona in 2015 (*N* = 2111)	
Iungman (2025) [41]	UGS (HIA)	Barcelona, Spain	≥ 20 years old, Barcelona residents in 2019 (*N* = 1,362,985)	
Wang (2022) [43]	Transport (HIA)	Gucun Township, Shanghai, China	Residing in Gucun Township, it is unclear if other eligible criteria were present (*N* = 289,500)	Territorial and Spatial Master Plan of Gucun Township (TSMPGT): An urban planning scheme including goals for 2035 in among other things: diversity, design, destination accessibility, distance to public transport. For example, distance to the nearest rail station is reduced from 2.3 to 1.38 km, and road network density increased from 4.46 to 6 km/km^2^
Laska (2019) [53]	Food (Natural experiment using a controlled before-after (difference-in-differences) design)	Minneapolis and St. Paul, Minnesota, USA	≥ 18 years old shopping at one of the stores. (Average per time point: Minneapolis *N* = 334, St. Paul *N* = 351)	Minneapolis Staple Foods Ordinance (MSFO): Mandates small food stores to stock minimum quantities of ten categories of healthy foods, including fruits, vegetables, 100% juice, whole grains, dairy, eggs, legumes, and meat and vegetable protein sources. Food stores are obliged to comply with the requirements to remain eligible for benefits, enforced by inspectors mandated to withdraw licenses
Hudak (2020) [52]	Food (Natural experiment using a controlled before-after (difference-in-differences) design)	North Carolina, USA	Shopping at one of the stores (near WIC stores *n* = 1470, near comparison stores *n* = 1530)	Special Supplemental Nutrition Program for Women, Infants, and Children (WIC): To receive benefits, WIC authorized food stores meet minimum inventory requirements. This includes fruits, vegetables, dairy, eggs, legumes, proteins, grains, and infant food. Among other things, WIC distributes food stamps to be spend in WIC stores, increasing store revenues. Food stores are obliged to comply with the requirements to remain eligible for WIC authorization
Minkler (2018) [54]	Food (Case study of policy implementation without statistical testing)	Tenderloin, San Francisco, USA	People who buy food in Tenderloin food stores (*N* = not described)	Healthy Retail San Francisco (HRSF): The HRSF incentivizes stores in food swamps to dedicate > 35% of selling space to healthy foods (such as fresh fruits and vegetables) and limiting alcohol and tobacco products to < 20%. In return, they receive benefits such as redesigns, store equipment, and training in business operations. Stores participate on a voluntary basis
Stevens (2011) [55]	Transport (natural experiment using cross-sectional evaluation)	Daybreak, Utah, USA	Being in fifth grade and living in a LEED_ND walkable community, mixed community or less walkable community (*N* = 187)	Leadership in Energy and Environmental Design Neighborhood (LEED_ND): This program awards points for pedestrian-friendly features, such as placing at least 50% of homes within Half a mile of a school, installing sidewalks of at least four feet wide, and positioning buildings close to the street
Hooper (2014) [50]	Mix: Transport, UGS (natural experiment using cross-sectional evaluation)	Perth, Western Australia	≥ 18 years, residing in one of the 36 developments for at least 2 years (*N* = 592)	Liveable neighborhood guideline (LNG): this is based on New Urbanism principles, promoting mixed-use, pedestrian -oriented, compact developments and human-scaled neighborhoods through community design, movement networks, lot layout, and public parkland, with norms for accessible neighborhood centers, minimum uses present in the center, and public transport access. For example, at least 15 dwellings/site should be within 400 m of major bus stops. Developers could voluntarily adopt the guidelines
Hooper (2015) [49]	Mix: Transport, UGS (natural experiment using cross-sectional evaluation)	Perth, Western Australia	≥ 18 years, residing in one of the 36 developments for at least 2 years (*N* = 664)	
Hooper (2018) [51]	Mix: Transport, UGS (natural experiment using cross-sectional evaluation)	Perth, Western Australia	≥ 18 years, residing in one of the 36 developments for at least 2 years (*N* = 664)	
Contardo-Ayala (2021) [45]	Mix: Food, UGS, transport (cross-sectional observational study)	Melbourne and Adelaide, Australia	≥ 18 years old residents, Melbourne: (*N* = 369) Adelaide: (*N* = 363)	20-minute neighborhood (20MN): a concept for living locally, enabling people to reach most of their daily needs, such as access to healthy food stores, recreational facilities, and health and education services within a 20-min nonmotorized transport trip from their home. The concept is promoted in planning strategies, but mandatory incentives are unclear
Oostenbach (2022) [46]	Mix: Food, UGS, transport (cross-sectional observational study)	Melbourne and Adelaide, Australia	≥ 18 years old residents, Melbourne: (*N* = 306) Adelaide: (*N* = 374)	
Oostenbach (2023) [48]	Mix: Food, UGS, transport (cross-sectional observational study)	Melbourne and Adelaide, Australia	Employed Melbourne or Adelaide residents ≥ 18 years old, (*N* = 378, 199 in 20MN, 179 in non-20MN)	
Lamb (2023) [47]	Mix: Food, UGS, transport (cross-sectional observational study)	Melbourne and Adelaide, Australia	≥ 18 years old residents, Melbourne (food sample *N* = 289, PA sample *N* = 337. Adelaide (food sample *N* = 353, PA sample *N* = 335)	
Yang (2022) [44]	Mix: Food, UGS, transport (cross-sectional observational study)	Melbourne and Adelaide, Australia	≥ 18 years old residents, Melbourne: (*N* = 637) Adelaide: (*N* = 692)	

*LNG* Liveable Neighbourhoods Community Design Guidelines, *WHO* World Health Organization, *UGS* Urban green space, *GA* Green Area, *MN* Minute Neighborhood, *HRSF* Healthy Retail San Francisco, *WIC* Special Supplemental Nutrition Program for Women, Infants, and Children, *TSMP* Territorial and Spatial Master Plan

Most applied studies evaluated voluntarily adopted policy standards, although legal enforcement was often unclear. Food environment interventions relied on voluntary store participation with mandatory compliance checks [52–54]. Risk of bias was moderate in 11 studies and serious in one (Supplementary Table 7). The additional quality appraisal found most HIAs used ERFs derived from meta-analyses [29–32, 34–36, 39–42], strengthening the underlying evidence (Supplementary Table 5), although the overall certainty of evidence was low to very low (Supplementary Table 6). Several HIAs modeled detailed and context-specific scenarios based on concrete urban or transport plans [37, 38, 41–43]. Sensitivity analyses examined alternative settings [29], standards [30, 31, 39], exposure measures [35, 37, 38], or ERFs [36, 40]. Overall, most studies showed good transferability between the target and source population, particularly regarding demographic and environmental comparability, and were sufficiently transparent and reproducible. One study demonstrated limited transferability because of substantial geographical differences and insufficient reporting of demographic characteristics [43].

As recommended by the PRISMA guidelines [21], we provide examples of studies that appeared to meet the inclusion criteria but were excluded, illustrating the application of our eligibility criteria. Two examples are Rojas-Rueda and Clockston (2021) [56] and Woodcock et al. (2014) [57]. Both evaluated the health effects of bicycle-sharing systems but were excluded for different reasons. The former projected shifts on transport mode share, representing a behavioral outcome rather than a transferable built environment standard itself. This review focused on policy standards that could be implemented across settings. The latter evaluated the London bicycle-sharing scheme and reported the number of bicycles and docking stations introduced. However, these were presented as absolute numbers rather than standardized measures (e.g., bicycles/1000 residents), making them context-specific to London and not transferable as generalizable policy standards.

### Study Results and Implementation Lessons

#### Urban Green Space

Prospective modeling studies predicted that UGS policy standards would reduce mortality, mental health conditions, and child behavior problems (Table 2). Across > 1000 European cities, the WHO universal UGS access standard projected 25 fewer deaths/100,000 (95% UI 19–38) when measured in normalized difference vegetation index (NDVI) [35]. Using % green area (%GA), the same study projected 11 fewer deaths/100,000 (95% UI 0–21). City-specific studies using %GA reported smaller mortality reductions: Bradford (4 deaths/100,000; 95% UI 0–8) [32], Vienna [31] and Madrid [29] (13 deaths/100,000; 95% UI 0–26), and Barcelona 21 deaths/100,000 (95% UI 0–43) in 2018 [29] versus 9 deaths/100,000 (95% UI 0–17) in 2012 [34]. The differing Barcelona estimates reflected different definitions of green space, with one study additionally including agricultural and seminatural areas [34]. The alternative standard of 9 m^2^ UGS/resident and the Viennese policy of 3.5 m^2^ UGS/resident projected mortality reductions of 8 and 2 deaths/100,000, respectively (both measured in %GA) [31]. The WHO access standard also projected 54 fewer cases/100,000 (95% UI 22–88) [33], and a 5.7% (95% UI 2.4–8.9; NDVI) to 9.6% (95%UI 2.0–17.8; %GA) projected reduction in childhood overweight/obesity [36].

**Table 2:** Summary table of outcomes reported by HIAs

Author	ERF	Context assumptions*	Methods and outcomes**	Baseline outcomes	Main findings	Reported implementation advice
Mueller (2017–1) [34]	Gascon (2016) [58] (meta-analysis) **Mortality** RR = 0.99 (95% CI: 0.98–1.01) per 10% increase in % Green Area (%GA)	- Providing 25.6% of UGS surface in all census tracts (CT) aligns to the WHO recommendation	The difference between recommended and baseline UGS levels (2012) was determined, ERF quantifying the association of UGS and mortality obtained from the literature, the RR and the PAF for UGS exposure difference was calculated as well as the outcomes (based on 2012 BHS): mortality rate	**Health outcomes** -Natural all-cause mortality rate: 1108 deaths/100,000*** **Environmental outcomes** - 31% of population without universal access to UGS	**Health outcomes** Annual reduction estimations if complied with the recommendation: - 9 deaths/100,000 (95% UI 0–17); 0.8% (0–1.5%) mortality reduction **** - 116 (95% CI 0–236) deaths prevented	Not reported
Mueller (2017–2) [33]	Triguero-Mas (2015) [72] (Cross-sectional study) **Depression** RR = 0.80 (95% CI: 0.71–0.91) per 0.08 normalized difference vegetation index (NDVI)	- Providing a mean NDVI score of 0.018 per CT aligns to the WHO recommendation	The difference between recommended and baseline UGS levels (2012) was determined, ERF quantifying the association of UGS and mortality/depression obtained from the literature, the RR and the PAF for UGS exposure difference was calculated as well as the outcomes (based on 2012 BHS): depression	**Health outcomes** -Depression Incidence rate: 895/100,000 **Environmental outcomes** −31% of the population without universal access to UGS	**Health outcomes** Annual reduction estimations if complied with the recommendation: *- 54 cases/100,000 (95% UI 22–88); 6.0% (2.5–9.8%) incidence reduction* - 740 cases of depression (95% CI: 295–1199) prevented	The authors advise implementing the WHO recommendation by implementing the Superblock model
Mueller (2018) [32]	Gascon (2016) [58] (meta-analysis) **Mortality** RR = 0.99 (95%CI: 0.98–1.01) per 10% increase in %GA	- Providing 25% UGS per area aligns to the WHO recommendation	The difference between recommended and baseline UGS levels (2012) was determined, the ERF quantifying the association of UGS and mortality was obtained from the literature, the RR and the PAF for UGS exposure difference was calculated as well as the outcomes (controlled for SEP, based on 2007–2010 born in Bradford study): mortality rate	**Health outcomes** - All-cause mortality rate was 1,114 deaths/100,000 **Environmental outcomes** −17.84% of the population without universal access to UGS	**Health outcomes** Annual reduction estimations if complied with standard: *- 4 deaths/100,000 (95% UI 0–8); 0.3% (0–0.7%)* - City wide: 16 deaths prevented (95% CI 0–32) **Differential effects Index of Multiple Deprivation** Projected mortality effect per deprivation quintile (1 = most deprived, 5 = least deprived) - Q1: 6/100,000, Q2: 4/100,000, Q3: 3/100,000, Q4: 1/100,000, Q5: 1/100,000 **Differential effects ethnicity** Projected mortality effect per LSOA level ethnicity - 80–100% nonwhite: 11/100,000, 60–80% nonwhite: 6/100,000, 40–60% nonwhite: 4/100,000, 20–40% nonwhite: 4/100,000, 0–20% nonwhite: 2/100,000	Given challenges in allocating large UGS in established cities and uncertainties about how they provide health benefits, enhancing existing greenery such as corridors, street trees, and pocket parks is crucial. Visual access to nature has been linked to stress reduction and restoration, making this approach more feasible and practical
Nguyen (2024) [36]	Ye(2022) [73] (meta-analysis) OR = 0.91 (95%CI 0.84–0.98) per 0.1 NDVI	- Providing 0.3111 NDVI or 25% GA aligns to the WHO recommendation	The difference between recommended and baseline percentage green area (%GA) or normalized difference vegetation index (NDVI) levels were determined, the ERF quantifying the association of UGS measures and overweight/mortality was obtained from the literature, the RR and the PAF were calculated at the ABS level for the outcome (controlled for age group, based on 2015 SIDIAP health record database): overweight/obesity rate	**Health outcomes** - 71,603 overweight/obese children (32.7%) **Per deprivation quintile** (Q1 = least deprived, Q5 = most deprived) Q1: 14,693 (25.8%) Q2: 13,729 (32.0%) Q3: 15,777 (33.8%) Q4: 14,219 (37.4%) Q5: 13,185 (38.3%) **Environmental outcomes** - 2015 average NDVI level 0.27 - 2015 average %GA 17.6%	**Health outcomes** Projected reduction under targeted **NDVI** levels: - 4094 (95% CI 1698–6379) overweight/obesity cases (5.7%; 95% *UI 2.4–8.9%)* Under targeted **%GA** levels: - 6853 (95% CI 1440–12,779) overweight/obesity preventable cases (9.6%; 95% *UI 2.0–17.8%)* **Differential effects deprivation index** Expected number of overweight/obesity attributed to UGS (Q1 = least deprived, Q5 = most deprived) **NDVI**: Q1: 771 (95% CI 334–1194), 4.8% *(95% UI 2.2–8.1%)* Q2: 1201 (95% CI 561–2007), 8.4% *(95% UI 4.1–14.6%)* Q3: 1216 (95% CI 579–1999), 7.7% *(95% UI 3.7–12.7%)* Q4: 398 (95% CI 188–691), 2.8% *(95% UI 1.3–4.8%)* Q5: 645 (95% CI 319–1071), 4.9% *(95% UI 2.4–8.1%)* **%GA**: Q1: 1443 (95% CI 264–2745), 9.8% *(95% UI 1.7–18.7%)* Q2: 1575 (95% CI 239–2995), 11.4% *(95% UI 1.7–21.8%)* Q3: 1863 (95% CI 334–3540), 11.8% *(95% UI 2.1–22.4%)* Q4: 937 (95% CI 165–1744), 6.6% *(95% UI 1.1–12.2%)* Q5: 987 (95% CI 170–1834), 7.5% *(95% UI 1.2–13.9%)*	Alternatives such as interior patio greening and green rooftops are being considered, although their accessibility remains uncertain. Additional proposals include more street trees, small gardens, pocket parks, vertical gardens, and equitably distributed green playgrounds in Barcelona and beyond
Barboza (2021) [35]	Rojas-Rueda (2019) [59] (meta-analysis) **NDVI**: HR = 0.96 on mortality risk per 0.1 unit increase in green exposure within 500 m buffer Gascon (2016) [58] (meta-analysis) **%GA**: RR = 0.99 (95% CI 0.98–1.01) per 10% increase in %GA	- Each residential grid cell needs to have 25%GA to provide universal access	The difference between recommended and current UGS levels for every grid cell in every city was determined, the ERF quantifying the association of both %GA and NDVI and mortality was obtained from the literature, the RR and the PAF for exposure difference was calculated as well as the outcomes: age-standardized mortality rate	**Health outcomes** Mortality rate 2015: 1,908,520 deaths (1128 deaths/100,000 inhabitants annually)	**Health outcomes** Annual reduction estimations if complied with recommendation: - NDVI: 22 deaths/100,000 (95% CI 17–33); 2.25% (95% CI 1.69%–3.36) 42,968 deaths (95% CI 32,296–64,177) - %GA: 11 deaths/100,000 (95% UI 0–21); 0.94% (95% CI 0%–1.87%) 17,947 deaths (95%CI 0–35,747)	Sensitivity analyses suggest exceeding WHO UGS recommendations prevent deaths. Cities are adopting plans to enhance urban quality and climate resilience by increasing UGS. Despite dense areas, opportunities for greening exist through repurposing industrial sites, adding green roofs, vertical gardens, street trees, pocket parks, green corridors, and reconfiguring road space
Iungman (2021) [29]	Gascon (2016) [58] (meta-analysis) **UGS** RR = 0.99 (95%CI 0.98–1.01) per 10% increase in %GA	- Providing 25% UGS per sub district aligns with the WHO recommendation	The difference between recommended and current UGS levels was determined, CT of 2018 was used for baseline mortality rate, the ERF quantifying the association of UGS and mortality was obtained from the literature, the RR and the PAF for UGS exposure difference was calculated, as well as mortality rates. The age-standardized mortality rate (ASAMR)/100,000 inhabitants was estimated at both the CT and city level, controlled for age, and deprivation index	**Health outcomes** Barcelona: 1082 deaths/100,000 Madrid: 977 deaths/100,000 - IRR lowest deprivation index quintile: 1.0 **Environmental outcomes** - Barcelona 35.3% UGS (2012), 95% of CT did not comply with standard - Madrid 41.5% UGS (2014), 83.5% of CT did not comply to standard	**Health outcomes** Annual reduction estimations if complied with the recommendation: Barcelona: *- 21 deaths/100,000 (95% UI 0–43); 1.9% (0–3.9%)* - 277 deaths (95% CI 0–581) - ASAMR = 19 (95% CI 0–41) deaths/100,000 prevented - IRR deprivation index quintile: very low: 1 (ref), low: 1.08 (*p* < 0.001), medium: 1.20 (*p* = 0.246), high: 1.21 (*p* = 0.009), very high: 1.42 (*p* = 0.008) Madrid: *- 13 deaths/100,000 (95% UI 0–26)*; *1.3% (0–2.6%)* - 337 deaths prevented (95%CI 0–684) - ASAMR = 12 (95%CI 0–24) deaths/100,000 prevented - IRR deprivation index quintile: very low: 1 (ref), low: 1.85 (*p* = 0.001), medium: 1.53 (*p* = 0.002), high: 1.47 (*p* = 0.045), very high: 1.73 (*p* = 0.058)	Previous research suggests UGS in low SES areas are often poorly maintained and underused due to associations with crime and vandalism. Consequently, the reception of UGS-related health benefits may be hindered. Therefore, attention should be given not only to the presence of UGS, but also to their quality and maintenance, to ensure positive health effects
Khomenko (2020) [31]	Gascon (2016) [58] (meta-analysis) **UGS** RR = 0.99 (95% CI 0.98–1.01) per 10% increase in %GA	- Providing 25% UGS per sub district aligns to the WHO recommendation	The difference between recommended and baseline UGS levels (2012) was determined, the ERF quantifying the association of UGS and mortality was obtained from the literature, the RR and the PAF for UGS exposure difference was calculated as well as the outcomes (controlled for sex and SES, based on 2017 PASTA Study): mortality rate	**Health outcomes** *- Natural all-cause mortality rate 1047 deaths/100,000* **Environmental outcomes** - City UGS: 49.6% - 78% did not comply with universal access standard - 39% without 3.5m^2^ UGS/resident (Viennese policy) - 55% without 9m^2^ UGS/resident (alternative WHO)	**Health outcomes** - Universal access to UGS (WHO): *13 deaths/100,000 (95% UI 0–26); 1.2% (0–2.5%)*; 190 deaths (95% CI 0–386) **Median attributable mortality per SES quintile** Very high SES quintile: 8/100,000 High SES quintile: 1/100,000 Medium SES quintile: 10/100,000 Low SES quintile: 14/100,000 Very low SES quintile: 13/100,000 - Viennese policy: *2 deaths/100,000 (95% UI 0–3); 0.2% (0–0.3%);* 26 deaths prevented (95% CI 0–52) - Alternative WHO standard: 8 *deaths/100,000 (95% UI 0–15); 0.7% (0–1.4%);* 114 deaths prevented (95%CI 0–230)	Given the challenges of expanding UGS in already developed urban areas, enhancing existing greenery such as street trees, green corridors, pocket parks, and small green areas becomes crucial. This form of greenery has been linked to various physical and mental health benefits
Dean (2024) [39]	Rojas-Rueda (2019) [59] (meta-analysis) **Mortality** HR: 0.96 (95% CI 0.94–0.97) per 0.1 NDVI within 500 m Paul (2020) [74] (large cohort study) **Stroke/dementia** HR: 0.97/0.96 (95% CI 0.96–0.98/0.95–0.98) per 0.12 NDVI within 250 m	- ERFs are applicable to arid or semi-arid areas, despite being based on other populations	ERFs were used to project block group all-cause mortality, stroke, and dementia implications of UTC policy goals translated to NDVI. This was done by applying their hazard ratios as a function of NDVI exposure. Mortality analysis controlled for age, sex, SES, smoking. Stroke/dementia analysis controlled for: age, sex, SES, comorbidities, population density, air pollution. Population-attributable health outcome counts for relevant age groups were estimated to calculate the attributable number of cases. The outcomes were: mortality and stroke/dementia incidence	Not reported	**Health outcomes** 20%/25% UTC in Denver/Phoenix annually prevents - 33/33 deaths/100,000 (95% UI 16–50/16–50) 200/368 deaths (95% UI 100–306/181–558)- 1/1 stroke case/100,000 (95% UI 1–2/1–2) 4/9 stroke cases (95% UI 2–7/5–14) - 2/2 dementia cases/100,000 (95% UI 1–4/1–3) 3/5 dementia cases (95% UI 2–4/3–8) Halfway scenario (16.5%/17% UTC): - 19/15deaths/100,000 (95%UI 9–30/7–22) 128/161 deaths (95% UI 62–197/80–244) - 1/1 stroke cases/100,000 (95% UI 0–1/0–1) 3/4 stroke cases (95% UI 1–4/2–7) - 1/1 dementia cases/100,000 (95% UI 1–2/1–2) 2/3 dementia cases (95% UI 1–2/1–4) **Difference between least and most Social Vulnerability Quartile** Denver 22 deaths/100,000 Phoenix 1 death/100,000	UTC benefits depend on factors such as tree species, age, humidity, and selection. Native trees, although having lower NDVI, offer drought tolerance, limited maintenance, and biodiversity. Careful selection and upkeep can reduce UTC-related issues such as allergens and obstructions
Kondo (2020) [30]	Rojas-Rueda (2019) [59] (meta-analysis) **HR** 0.96 (95% CI 0.94–0.97) on all-cause mortality per 0.1 unit increase in NDVI within 500 m	- the ERF works the same for all SES groups - TCC goals assume TCC remains steady and will only increase over time	A HIA was conducted to determine the annual premature mortality burden (based on 2015 vital statistics report) due to projected changes in TCC (baseline 2014, projections for 2025). The assessment utilized ERFs and modeled TCC changes at CT level. The study estimated (controlled for CT SES): mortality rate	**Health outcomes** - Mortality rate (2015) 887 deaths/100,000 **Environmental outcomes** - The average percent TCC in CT City-wide: 20.3% Lower SES: 16.6% Higher SES 24.1%	**Health outcomes** Annually prevented premature deaths 30% TCC - City-wide 403 (95%CI 298–618); 2.9% - Low SES CT 244 (95%CI 180–373); 3.6% - High SES CT159 (95%CI 11–244); 2.4%	In low SES areas, greening projects may introduce gentrification and cause concern among residents due to past neglect in maintenance. To ensure economic diversity, tree planting initiatives in low SES communities may require additional policies, e.g., preserving affordable housing or supporting at-risk tenants. To reach the 30% TCC goal, tree planting programs must encompass not only public areas, but also private spaces. However, challenges persist in gaining resident participation and ensuring long-term participation
Iungman (2023) [40]	Masselot (2023) [75] (meta-analysis) **UGS**-related temperature RR depends on the age group and percentile	Not reported	The effect of increasing tree coverage to 30% (per 250 × 250 m grid cell) was analyzed, and scenarios for 40% and 25% were also calculated. City-specific and age group-specific ERFs were retrieved. Daily baseline temperature exposure levels were determined and the PAF for each daily mean and age group was calculated, as well as the premature mortality burden (controlled for age)	**Health outcomes** - 555,215 all-cause mortality (2015), of which 128,269 (23.1%) in summer **Environmental outcomes** - Mean maximum temperatures: 22.7–36.8 °C - Mean city TCC: 14.9% (SD 13.9)	**Health outcomes** Premature heat-related deaths prevented by increasing TCC to: - 30% TCC: 2,644 (95%CI 2,444–2,824); 1.84% (1.69%–1.97%) - 40% TCC: 3,727 (95%CI 3,462–3,992)2.58% (2.38%–2.76) - 25% TCC:2,092 (1,933–2,241) 1.46% (1.34%–1.57%)	Groups of trees rather than isolated ones may result in synergic effects, improving the trees’ cooling capacity. Meeting the goal is not possible for all areas, therefore the intervention scale should vary. The results suggest a linear association for TCC and cooling effects, facilitating adaptable planning. The 30% tree coverage target is encouraged, but a 25% target may be more feasible for compact cities, combined with other cooling strategies, such as green roofs
Mueller (2020) [42]	Gascon (2016) [58] (meta-analysis) **UGS** RR = 0.99 (95%CI 0.98–1.01) per 10% increase in %GA **PA** RR = 0.81(95%CI 0.76–0.85) 11vs0 MET h/week **NO2** RR = 1.02(PI 0.99–1.06) per 10 μg/m^3^ **Noise** HR = 1.04 (95%CI 1.02–1.06) per 10 dB Lden **Heat** RR = 1.19 (95%CI 1.16–1.23) Tₚ₉₉ vs Tₚ₇₄	- The ERF used for PA is curvilinear, the ERFs used for air pollution, noise, UGS, and heat are linear	The baseline situation (2010) was compared with the counterfactual scenario (i.e., implementing 503 SBs). ERFs to quantify the strength of association of the exposures and mortality were obtained from the literature. The exposure data was combined with the ERFs, population data and mortality statistics to calculate PAFs to quantify the SB scenario outcomes (based on 2016/2017 BHS): mortality rate	**Health outcomes** City wide annual (mean): -Annual natural-cause mortality rate was 1144 deaths/100,000 **Environmental outcomes** −1,190,000 car trips/weekday - NO2 level: 47.2 µg/m3 - traffic noise levels 54.2 dB Lden - 132 days at least one SB exceeds MMT of 21.5 °C - Gridded area: mean UGS 6.5%	**Health outcomes** Annual reduction estimations after implementation of 503 SBs city wide: - *51 deaths/100,000 (95% UI 18–84); 4.5% (1.6–7.3%)* - 667 premature deaths (95% CI 235–1098) **Environmental outcomes** - 962,000 car trips/weekday (− 228,000 car trips/weekday; − 19.2%) - NO2 reduced to 35.7 µg/m^3^ (− 11.5 µg/m^3^; − 24.3%) - Traffic noise levels reduced to 51.3 dB Lden (− 18.5 dB Lden; − 5.4%) - 116 days at least one SB exceeds MMT of 21.5 °C (− 16 days; − 12%) - UGS increased to 19.6% (+ 13.1%)	It is essential to improve the suburban commuter network alongside implementing the SB model, because a significant portion of trips are made by private motorized transport. The SB model poses the risk of gentrification, where improvements in underserved areas attract wealthier residents, driving up rents and displacing current inhabitants To prevent unwanted traffic relocation to disadvantaged areas outside SBs, additional measures discouraging private motorized transport are necessary, such as reducing on-road parking and congestion charges. To mitigate these risks, it is crucial to implement the SB model consistently and equitably
Vidal-Yañez (2023) [38]	Triguero-Mas (2015) [72] (cross-sectional) Per 0.08 NDVI increase **Self-perceived poor mental health**: RR = 0.79 **Sedative use:** RR = 0.88 **Antidepressant use**: RR = 0.80 **Visits to mental health specialists:** RR = 0.80	- ORs are used to approximate RRs thanks to Low occurrence of mental health outcomes (< 10%)	The HIA process involved calculating the difference between baseline and Eixos Verds UGS (%GA and NDVI) exposure levels for each CT, and using the ERF to calculate the RR and PAF for exposure difference as well as the mental health outcomes: self-perceived mental health, sedative use, antidepressant use, mental health specialists’ visits	**Health outcomes** Prevalence rate/100,000 - Self-perceived poor mental health: 18,095 - Sedative use: 9460 - Antidepressant use 8095 - Visits to mental health specialists: 10,168	**Health outcomes** Prevented annual reduction estimations if Eixos Verds is implemented: −31,353 (95% CI 18,126–42,882) cases of self-rated low mental health;14.0% (95% CI 8.1–19.2%)) −9476 (95% CI 802–16,391) cases of sedative use;8% (95% CI 0.7–14.0%) −13,375 (95% CI 6107–19,184) cases of antidepressant use; 13.4% (95% CI 6.1–19.2%) −16,800 (95% CI 6828–25,700) visits to mental health specialists;13.4% (95% CI 5.4–20.5%) **Environmental outcomes** - Increase of 5.7%GA and 0.06 NDVI level	After Eixos Verds implementation, Barcelona will have 17% UGS, below the recommended 25%. A more ambitious scenario with a 10-m street greening extension would raise coverage to 21%, closer to the target. The Eixos Verds and SB models pose risks such as traffic disruption, parking loss, and gentrification. To mitigate, green interventions should be evenly spread, with complementary policies for transport, low-emission zones, and gentrification measures
Opbroek (2024) [37]	Amoly (2014) [76] (cross-sectional) Per IQR NDVI incr - **Total difficulties score** SDQ − 3.8% (95% CI − 6.4; − 1.2) - **Hyperactivity/inattention score** SDQ − 4.5% (95% CI − 7.4; − 1.6)	- Street greening, at surface or canopy level, would extend to a maximum of 10 m street width	The HIA process involved calculating the difference between baseline and Eixos Verds UGS (%GA and NDVI) exposure levels for each CT, and using the ERF to calculate the RR and PAF for exposure difference as well as the child behavioral outcomes: total difficulties and hyperactivity/inattention scores	**Environmental outcomes** - CT mean of 4.2 percentage green area (%GA). Baseline CT mean NDVI level was 0.262	**Health outcomes** Estimations one year after implementation of the plan: SDQ’s total difficulties scores − 5.2% (IQR 6.5%; 95% CI 0–14.8%) Hyperactivity/inattention scores − 6.1% (IQR 7.7%; 95% CI 0–17.3%) **Environmental outcomes** - CT mean increase of 6.9%GA and 0.065 NDVI	To ensure equitable benefits for children’s development, the Eixos Verds should consider child density and socioeconomic factors. UGS proxies (%GA, NDVI) do not reflect quality or children’s actual use, which affects activity levels and access
Iungman (2025) [41]	Rojas-Rueda (2019) [59] (meta-analysis) **UGS** (NDVI) HR = 0.96 (95% CI 0.94–0.97) Masselot (2023) [75] (meta-analysis) **Temperature** RR depends per age group and percentile	- Streets ≥ 20 m wide; increase 15% in UGS; 0.09 trees/m - Streets ≤ 20 m wide, increase 7% in UGS; 0.035 trees/m - Axes ≥ 30 m wide, increase 7% UGS; 0.13 trees/m	The HIA process involved calculating the difference between baseline and Eixos Verds UGS (NDVI) and separate temperature exposure levels for each CT and using the ERF to calculate the RR and PAF for exposure difference as well as the preventable premature mortality burden (temperature analysis controlled for age)	**Health outcomes** Mortality rate 2019: 1054/100,000 **Environmental outcomes** - Average NDVI: 0.27 (IQR, 0.22–0.30) per CT - Mean temperature 26.91 °C (min 24.86, max 27.71) per CT	**Health outcomes** Prevented annual reduction estimations if Eixos Verds is implemented: - NDVI: 178 deaths (95% CI 116–247); 13/100,000 (95% CI 9–19); 1.2% (95% CI 0.8–1.7%) - Temperature: 5 deaths (95% CI 4–6)*; 0.4/100,000 (95% UI 0.3–0.4); 0.03% (95% UI 0.03–0.04%)* **Environmental outcomes** - Mean NDVI increased to 0.286 (IQR: 0.256–0.304), average increase of 0.019 (IQR: 0–0.04) per CT - Mean temp reduction 0.05 °C (IQR: 0.01–0.07) per CT	The concentration of greenness lies towards the outskirts, resulting in lower UGS availability in the central, densely populated areas. With the Eixos Verds, distribution improves. More ambitious strategies are required to effectively reduce ambient temperatures. Considering extensive areas of roofs suitable for greening compared to streets, this could yield more substantial results
Wang (2022) [43]	Kyu (2016) [77] (meta-analysis) **CVD morbidity** risk related to insufficient PA (< 600 MET min/week): RR = 1.032 **CVD morbidity risk** related to 10 ug/m3 increase in PM2.5: RR = 1.11	Not reported	The HIA process involved calculating the changes in exposure levels between the current situation (2019) and the planning scenario (2035) and using the ERF to calculate the RR and PAF under varying exposure levels of the following outcomes: % changes in traffic-related PM2.5 concentration, weekly commuting walking time, CVD incidence	**Health outcomes** - Average commuting walking time: 17.4 min/day (487 MET minutes/week)	**Health outcomes** Implementing the TSMP projects to: - Lower CVD morbidity by 5.3% (3.2% from increased physical activity and 2.2% from reduced air pollution) Improved expected health behaviors: - Increase average commuting walking by 7.7 min/day (215 MET minutes/week) **Environmental outcomes** - Reduce vehicle miles traveled by 22.7%, reduce traffic-related PM2.5 by 2.1 µg/m^3^	Not reported

*CT* census tract, *ERF* exposure response function, *WHO* World Health Organization, *BHS* Barcelona Health Survey, *PA* physical activity, *CI* confidence interval, *SES* socioeconomic status, *SEP* socioeconomic position, *OR* odds ratio, *MMT* minimum mortality temperature, *UGS* urban green space, *SB* superblock, *TCC* tree canopy coverage, *IQR* interquartile range, *NDVI* normalized difference vegetation index, *HR* hazard ratio, *ASAMR* aged standardized attributable mortality rate, *CVD* cardiovascular disease, *RR* relative risk, *GA* green area, *UI* uncertainty interval, *SDQ* Strengths and Difficulties Questionnaire, *HIA* Health Impact Assessment, *PAF* population attributable fraction, *RR* relative risk, *LSOA* lower super output area, *ABS* Area Basica Sanitaria (basic health zone), *PASTA* physical activity through sustainable transport approaches

*In addition to context-specific assumptions, key assumptions related to PAF include: a causal relationship between risk factors and disease, immediate risk reduction upon removal of exposure, and independence of the considered risk factors from other disease-influencing factors [78]. HIAs also rely on reliable data and robust analysis, aiming to provide recommendations based on interpretation of evidence rather than absolute truths [79]. Other general assumptions include: the policy is implemented fully and without restrictions; health benefits occur immediately unless otherwise specified; ERFs are linear unless stated otherwise; and the population size and composition remain constant in the counterfactual scenario, even if projecting into a future year; RRs are uniform across individuals unless otherwise specified

**If no control variables are mentioned, the analysis did not have any control variables

***Per 100,000 always means per 100,000 persons

****Results in italic are calculated by us (See Supplementary Table 8) to harmonize outcomes and facilitate comparison across studies

Several studies modeled tree canopy cover (TCC) goals. Across 93 European cities, achieving 30% TCC was projected to prevent 1.8% (95% CI 1.7–2.0) of annual heat-related deaths; 40% and 25% TCC corresponded to projected reductions of 2.6% (95% CI 2.4–2.8) and 1.5% (95% CI 1.3–1.6), respectively [40]. In Philadelphia, 30% TCC was projected to prevent 403 (95% CI 298–618) all-cause deaths annually [30]. In Denver and Phoenix, 20% and 25% TCC were projected to prevent 33 deaths (95% UI 16–50), 1 stroke case (95% CI 1–2), and 2 dementia cases (95% CI 1–3) per 100,000 annually in each city [39]. Intermediate scenarios (16.5% and 17%) projected 19 (95% CI 9–30) and 15 (95% CI 7–22) fewer deaths/100,000, respectively, together with 1 fewer stroke (95% CI 0–1) and 1 fewer dementia (95% CI 1–2) case/100,000.

The Eixos Verds plan, converting one-third of streets into green corridors (measured in NDVI), was projected to reduce annual mortality by 13/100,000 (95% CI 9–19) [41], prevent 14% (95% CI 8.1–19.2) of poor self-perceived mental health cases [38], and improve child behavior through lower total difficulties (−5.2%; 95% CI 0–−14)) and hyperactivity/inattention scores (−6.1%; 95% CI 0–−17) [37]. No studies evaluated the health effects of applied UGS policies.

Studies emphasized prioritizing park quality and maintenance [29], while expanding pocket parks, street trees, and green corridors [31, 32, 35]. Several warned policymakers of unintentional gentrification [30, 38] and recommended consistent and equitable implementation [37, 42]. In dense cities, greening opportunities include repurposing industrial sites, reconfiguring road space, and adding vertical gardens or green roofs [35, 40, 41]. One study noted that groups of trees provide greater cooling than individual trees [40]. Finally, commonly used UGS proxies (%GA; NDVI) capture vegetation quantity but not quality or use [37]. Because all UGS studies were prospective modeling studies, no unplanned effects were reported.

#### Food Environment

Two of three studies on stocking requirements were associated with increased healthy food sales [52, 54] (Table 3). The Special Supplemental Nutrition Program for Women, Infants, and Children (WIC) was associated with increased WIC food sales by 16.3 units/week/1000 people [54], while Healthy Retail San Francisco (HRSF) reported 35% higher fresh produce sales across four stores [54]. The Minneapolis Staple Foods Ordinance (MSFO) study found no significant changes in calories purchased or Healthy Eating Index (HEI)−2010 score compared with controls over time [53].

**Table 3:** Summary table of outcomes of nonrandomized evaluations of implemented policies

Author	Methods and outcomes*	Baseline/control group outcomes	Main findings	Unplanned/secondary effects	Reported implementation lessons learned
Laska (2019) [53]	Stores in Minneapolis (*n* = 84) and St. Paul (n = 71) (controls) were compared using bivariate Chi-square tests for percentages and general linear models for means. The observations were: 2014 prepolicy, 2015 implementation, 2016 enforcement, and 2017 monitor. The overall time x city interaction was done to assess the effect over all times (controlled for neighborhood race/ethnicity, repeated measures): HEI score, calories purchased	**Health outcome** - HEI-2010 score**: Minn.: 30.7, St. Paul: 29.9 - Calories purchased: Minneapolis: 962, St. Paul: 1421 **Environmental outcomes** - Met all ordinance standards: Minn.: 7.4%, St. Paul: 0.4% - HFS score***: Minn.: 10.6, St. Paul: 8.7	**Health outcomes** - HEI score*: *p* (time x city) = 0.69 - Calories purchased: *p* (time x city) = 0.76 **Environmental outcomes** - Met all ordinance standards: *p* (time x city) = 0.57 - Healthy Food Supply score**: p (time x city) = 0.99	Not reported	To increase store compliance, sufficient enforcement is needed, including inspections consequences after violations. Alongside minimum stocking requirements, additional marketing strategies for placement, pricing and promotion of healthy food are necessary for a healthier food environment
Hudak (2020) [52]	Weekly sales data over 19 months provided by 8 WIC authorized and 8 sales-matched non-WIC authorized discount variety stores were analyzed in a difference-in-differences regression (controlled for SES, income, race, census-block population, vehicle access, number of near SNAP- stores) to evaluate how WIC authorization affected weekly:- sales WIC foods, sales non-WIC foods	Not reported	**Health outcomes** Difference-in-difference estimator: - 30.9 (95%CI 14.1–47.9) units/1000 people increase in sales of WIC foods/week - 16.3 (95%CI 11.1–21.5) units/1000 people increase in sales of WIC foods/week/1000 persons	4.3% of the WIC items and 44.3% of the new items were purchased with WIC vouchers. Sales for WIC stores increased with $74.6 (95%CI 39.9; 109.3) for WIC foods and $32.3 (95%CI −29.8;94.4) for non-WIC foods	Monitoring and approving WIC vendors demands resources from WIC State Agencies, potentially diverting funds from vital programs such as breastfeeding peer support. Therefore, the costs of overseeing additional vendors must be carefully balanced against the benefits of broader access to WIC foods
Minkler (2018) [54]	One year after initiation of the program, point-of-sale data were collected monthly from the four out of the five HRSF stores and analyzed to identify any fluctuations in produce and sales over one year. It is unclear if any statistical technique was used. Stores were assessed with a star rating system (1–4 stars) by “food justice leaders” (hired trained residents) between 2013 and 2017 with report cards. Outcomes were: POS data of healthy food	**Health outcomes** - Analyzed stores (*n* = 4) in 2013: on average 5.299 units fresh produce sold **Environmental outcomes** - Neighborhood (35 stores) in 2013 - 77% stores: low score (1–2 star); 1 store: highest rating (4 stars) - 42% sold no fresh food	**Health outcomes** Analyzed stores (*n* = 4) 12 months after initiation - Four stores: 7.174 units fresh produce sold (+ 35%) - One store: 0% change in tobacco sales **Environmental outcomes** Neighborhood (35 stores) in 2015 - 52% of the stores received low score (1 or 2 stars) Neighborhood (35 stores) in 2017; 49% of the stores received low scores (1 or 2 stars); 4 stores with the highest rating (4 star)	A ripple effect was reported: nonparticipating stores improved their store environment. Store managers’ reasoning included staying competitive, hoping to get into HRSF, and as “customers deserve it”	The program distinguishes itself with involving residents, organizations, policymakers, and academics. They engaged in advocacy efforts, capacity building, and community-led research. Key strategies included involving store associations in policies, recruiting community members for participatory research, and leveraging windows of opportunity
Stevens (2011) [55]	Participants (2007) sampled from two schools and lived in a LEED_ND walkable community, mixed community (lived in a less walkable area but attended a walkable school) or less walkable community. ANCOVAs were conducted (controlled for parental walking preference, in-school MVPA): MVPA disparities during school days based on reported walking to/from school, excluding weekends	**Health outcomes** Mean MVPA minutes/day for children from less walkable communities, attending schools in less walkable neighborhoods (control): In the 0.5 h before school: 5.07 In the 0.5 h after school: 6.35	**Health outcomes** Mean MVPA min/day for children in LEED_ND communities attending school in LEED_ND neighborhoods: - 0.5 h before school: 6.93 min (+ 1.86; *p* < 0.10) - 0.5 h after school: 9.13 min (+ 2.78; *p* < 0.05) Mean MVPA min/day for children from less walkable communities attending schools in LEED_ND neighborhoods (mixed): - 0.5 h before school: 6.72 min (1.65; *p* < 0.05) - 0.5 h after school: 10.38 min (+ 4.03; *p* < 0.05)	Not reported	Not reported
Hooper (2014) [50]	Participants completed the RESIDE survey (2009), at least two years after relocating to the new neighborhoods. K-means cluster analyses clustered developments by the similarity of design features implementation. Logistic regression assessed the association, per 10% increment of policy compliance and per identified cluster, with the likelihood of undertaking (controlled for age, gender, education, children at home, stage of construction, development size, residential self-selection (RSS)): any transport/recreation walking, 60 min transport/recreation walking	**Health outcomes** Cluster analysis: ORs for cluster types and walking in the neighborhood: - Cluster 1 (C1): Scored bad overall (control group, 5 developments, *n* = 47) Cluster 1 (C1) Any WT: 1.0 C1 ≥ 60 min WT: 1.0 C1 Any WR: 1.0 C1 ≥ 60 min WR: 1.0 **Environmental outcomes** - Compliance scores ranged 27–53% (mean 45%) for conventional developments	**Health outcomes** Cluster analysis: ORs for cluster types and walking in the neighborhood compared to C1 - C2: best movement network and lot layout (23 developments, *n* = 436) Any WT 1.54 (95% CI 0.79–2.99), ≥ 60 min WT 1.74 (95% CI 1.22–2.48); Any WR 1.29 (95% CI 0.56–2.93), ≥ 60 min WR 2.05 (95% CI 1.46–2.88) - C3: best public parkland (5 developments, *n* = 47) Any WT 1.43 (95% CI 0.53–3.91), ≥ 60 min WT 3.53 (95% CI 2.02–6.17); any WR 0.83 (95% CI 0.31–2.22), ≥ 60 min WR 3.37 (95% CI 1.98–5.74) - C4: best community design (3 developments, *n* = 107) Any WT 2.64 (95% CI 1.38–5.06), ≥ 60 min WT 0.96 (95% CI 0.68–1.36); any WR 1.98 (95% CI 1.09–3.61), ≥ 60 min WR 0.96 (95% CI 0.75–1.22) - Compliance analysis: ORs of walking in the neighborhood for 10% increases in compliance scores Any WT: 1.53 (95% CI 1.13–2.08), ≥ 60 min WT: 1.14 (95% CI 0.70–1.88); any WR: 0.97 (95% CI 0.73–1.30), ≥ 60 min WR: 0.97 (95% CI 0.74–1.27) **Environmental outcomes** - Compliance scores ranged 30–55% (mean 47%) for livable developments	Adoption of the policy principles diffused, many of the conventional developments also included LNG requirements. General compliance to policy was low	Flexible permitting practices partly explain the low level of compliance, as none of the projects were fully completed, and contribute to the lack of discernible differences between LNG and conventionally designed neighborhoods. Challenges stemmed from deficiencies in the structure and content of LNG documents. Conflicts arose between LNG policy and established regulations governing engineering policy standards. Administrative and political hurdles, as well as a lack of incentives for compliance, hampered developers’ ability and willingness to comply with LNG requirements
Hooper (2015) [49]	Participants completed the RESIDE survey (2009), at least 2 years after relocating to the new neighborhoods. K-means cluster analyses clustered developments by the similarity of design features implementation. Logistic regression assessed the association, per 10% increment of policy compliance and per identified cluster, with the likelihood of undertaking (controlled for age, gender, education, children at home, stage of construction, size of development, RSS): any transport/recreational/total walking, 60 min transport/recreational/total walking, 150 min total walking	**Health outcomes** ORs of walking in the neighborhood for residents - C1: Scored bad overall (control group, 5 developments, *n* = 47) Any WT: 1 ≥ 60 min WT: 1 Any WR: 1 ≥ 60 min WR: 1 Any TW: 1 ≥ 60 min TW: 1 ≥ 150 min TW: 1	**Health outcomes** ORs walking in the neighborhood for residents in clusters C2-C4 compared to C1 - C2: best movement network and lot layout (23 developments, *n* = 436) Any WR: 1.74 (95% CI 1.22, 2.48), ≥ 60 min WR: 2.05 (95% CI 1.46, 2.88) - C3 best public parkland (5 developments, *n* = 47) Any WR: 3.53 (95% CI 2.02, 6.17), ≥ 60 min WR: 3.37 (95%CI 1.98, 5.74) Any TW: 2.35 (95% CI 1.36, 4.09) - C4 best community design (3 developments, *n* = 107) Any WT: 2.64 (95% CI 1.38, 5.06), ≥ 60 min WT: 1.98 (95% CI 1.09, 3.61) Any TW: 1.71 (95% CI 1.44, 2.03), ≥ 60 min TW:1.77 (95%CI 1.14, 2.76) ≥ 150 min TW: 1.47 (95% CI 1.15, 1.86)	Not reported	The cluster analysis suggests multiple design features are needed to support recreational walking. Achieving cohesive new developments is challenging when adjacent projects are managed by different developers with planning requirements. Smaller subdivisions should be regionally planned to ensure timely and equitable provision of services. Responsibilities and contributions from governments and developers must be clearly identified
Hooper (2018) [51]	Participants completed the RESIDE survey (2009), at least two years after relocating to the new neighborhoods. K-means cluster analyses clustered developments by the similarity of design features implementation. Logistic regression assessed the association, per 10% increment of policy compliance and per identified cluster, with the likelihood of (controlled for Age, gender, education, number of children at home, construction stage, development size, RSS, marital status, sense of community, design elements, minutes walking): psychological distress, sense of community	Compliance scores ranged from 30–55% (average 47%) for livable developments and 27–53% (average 45%) for conventional developments	**Health outcomes** ORs (95%CI) per 10% increased policy compliance, separate models: Low psychological distress: Total LNG compliance: 1.11 (1.01–1.23), community design: 1.08 (1.04–1.11), movement network: 1.27 (0.93–1.74), lot layout: 1.02 (0.9807–1.06), public parkland: 0.99 (0.86–1.10) ORs per 10% increase compliance, entered in the same model: Low psychological distress: Community design: 1.10 (1.02–1.18), movement network: 1.16 (0.71–1.91), lot layout: 0.95 (0.89–1.01), public parkland: 0.98 (0.86–1.08) High sense of community: Community design: 1.05 (1.03–1.14), movement network: 0.98 (0.62–1.53), lot layout: 0.96 (0.87–1.05), public parkland: 1.00 (0.89–1.13)	Adoption of the policy principles diffused, many of the conventional developments also included LNG requirements	The LNG developments included comprehensive goals such as developing train stations and ample local employment opportunities, which were not yet realized at the time of evaluation. Developing and assessing the full impact of a functioning transit-oriented neighborhood with sufficient destination density takes longer than the evaluation period of this study
Contardo-Ayala (2021) [45]	It was examined if those residing in a 20MN compared with non-20MN have higher odds of walking for transport and for recreation, and if they spend more time walking for transport and for recreation. Logistic regression models assessed if walking odds differed by neighborhood types (20 MN and non-20MN). Two-part models estimated mean weekly minutes of (controlled for age, gender, education, RSS): walking for transport, walking for recreation	**Health outcomes** - Mean time WT (min/w) Melbourne non-20MN: 41.2 95% CI 32.7–49.7) Adelaide non-20MN: 43.9 (95% CI 32.9–54.8) - Mean time WR (min/w) Melbourne non-20MN: 141.0 (95% CI 122.6–159.4) Adelaide non-20MN: 134.3 (95% CI 119.0–149.5)	**Health outcomes** - Mean time WT (min/w) Melbourne 20MN: 82.5 min/week (95% CI 65.3–99.7) Adelaide 20MN 49.2 min/week (95% CI 39.5–58.8) - Any WT (vs non-20MN) Melbourne 20MN: OR 4.24 (95% CI 2.38–7.56) Adelaide 20MN: OR 1.31 (95% CI 0.80–2.13) - Mean time WR (min/w) Melbourne 20MN: 129.8 (95% CI 106.8–152.7) Adelaide 20MN: 140.0 (95% CI 120.2–159.9) - Any WR (vs non-20MN) Melbourne20MN: OR 1.6 (95% CI 0.88–2.76) Adelaide 20MN: OR 1.04 (95% CI 0.59–1.86)	Not reported	Not reported
Oostenbach (2022) [46]	Associations are examined in the frequency and location of eating out behaviors and home food delivery in 20MNs and non-20MNs in both cities. Ordinal regression models were fitted for: Frequency of visiting Major fast-food chains. Analysis controlled for age, gender, education, neighborhood SES, manage income, children at home, RSS	**Health outcomes** Fraction of non-20MN participants visiting food outlets at least once p/w - Melbourne: fast-food chains 14.7%/12.4% - Adelaide: fast-food chains 12.1%/11.9%	**Health outcomes** Models for frequency of visits to food outlets, takeaway (delivery) and N types of weekly frequented outlets for 20MN residents (vs non-20MN) - Melbourne Major fast-food chains: OR 0.86 (95%CI 0.45–1.65) - Adelaide Major fast-food chains: OR 1.40 (95%CI 0.77–2.55)	Not reported	Not reported
Oostenbach (2023) 2 [48]	It was examined how work hours and commute time relate to consumption and whether 20MNs moderate this association. IRR and OR of food consumption outcomes/h increase in work/commute hours among those employed were calculated for 20MN and non-20MNs. Poisson regression was used for: daily serves of fruit/veg, n discretionary food types; ordinal regression for freq. of: consumption of snacks, soft drinks. Controlled for age, neighborhood SES, living status, gender, children at home, city	**Health outcomes** The estimate is the change in IRR/OR per h increase in work/commute hours in non-20MN: Daily fruit IRR 1.0 (95% CI 0.99–1.0); daily veg IRR 1.0 (95% CI 0.99–1.01) Snacks OR 1.01 (95% CI 1.0–1.03); soft drinks OR 1.01 (95% CI 0.98–1.03)	**Health outcomes** The estimate is the change in IRR/OR per h increase in combined work/commute hours in 20MN: Daily fruit IRR 1.0 (95% CI 0.99–1.01) Daily vegetables IRR 1.0 (95% CI 1.0–1.01) N types of discretionary food consumed weekly IRR 1.0 (95% CI 1.0–1.01) Snacks OR 1.00 (0.99–1.02) Soft drinks OR 1.02 (0.99–1.04)	Not reported	Not reported
Lamb (2023) [47]	Ordinal regression was used to assess the effect on diet outcomes and self-rated health. Two-part models were fitted to walking outcomes. Poisson regression was used to analyze the n of activities undertaken. Interactions between 20MN and SES were included in each model. Outcomes assessed were (controlled for age, gender, neighborhood and individual SES, living status, children at home, city, RSS): daily serves of fruits/veg, WT, WR, n weekly exercise, self-rated health		The study reported numerous detailed findings without prioritizing key results. Overall, there was little evidence that the associations of living in a 20MN and dietary behaviors, physical activity, or self-rated health varied by area-level or individual SES. Refer to the original paper for the full set of findings	Not reported	Not reported
Yang (2022) [44]	In each city OLS linear regression was used to examine associations between having a 20MN and BMI between Oct 2018 and May 2019. Moran’s *I* test was used to assess spatial autocorrelation in the residuals of these models. To account for residual spatial autocorrelation in the OLS regression models, spatial error regression models were fitted to assess (controlled for age, gender, SES, ability to manage household income, survey type, spatial autocorrelation): BMI	**Health outcomes** - Melbourne non-20MN Adjusted: BMI 22.86 kg/m^2^ - Adelaide non-20MN Adjusted: BMI 25.44 kg/m^2^	**Health outcomes** - Melbourne 20MN: BMI 22.64 kg/m^2^ (95% CI 24.27, 25.55), 0.22 kg/m^2^ (95%CI − 0.48–0.93) lower than non-20MNs - Adelaide 20MN: BMI 24.73 kg/m^2^ (95% CI 23.04, 26.41), 0.71 kg/m^2^ (95% CI − 0.05–1.46) lower than non-20MNs	Not reported	Not reported

*WT* walking for transport, *WR* walking for recreation, *TW* total walking, *OR* odds ratio, *IRR* incidence risk ratio, *CI* confidence interval, *MN* minute neighbourhood, *MVPA* moderate-to-vigorous physical activity, *SNAP* Supplemental Nutrition Assistance Program, *HEI* Healthy Eating Index, *HFS* Healthy Food Supply, *STORE* STaple foods ORdinance Evaluation, *ProjectPLAN* Places and Locations for Activity and Nutrition study, *RSS* Residential self-selection

*If no control variables are mentioned, the analysis did not have any control variables

**The HEI-2010 score (Healthy Eating Index) is a measure of diet quality in terms of conformance to the Dietary Guidelines for Americans (DGA). It consists of 12 components each with a standard for scoring, totaling up to 100 (minimum score = 0)

***Healthy Food Supply (HFS) score summarizes availability, price, quality, and variety of available foods. The HFS score ranged 0–31, where higher scores indicate healthier stores

Successful implementation depended on adequate enforcement [53], supplemented by marketing strategies, store union involvement, and community engagement [53, 54]. Reported unplanned effects included increased revenues in participating stores [52] and higher healthy food sales in nearby non-participating stores [54].

#### Transport Environment

A HIA on the Territorial and Spatial Master Plan of Gucun Township (TSMPGT) in Shanghai projected a 3.1% reduction in cardiovascular disease morbidity by 2035, primarily through increased walking (+ 214.75 MET-minutes/week) (Table 2) [43].

Among applied policies, implementation of the Leadership in Energy and Environmental Design Neighborhood (LEED_ND) program was associated with children accumulating 2–4 additional minutes/day (*p* < 0.05) of moderate-to-vigorous physical activity (MVPA) before or after school compared to children in conventional neighborhoods (Table 3) [55]. No implementation lessons or unplanned outcomes were reported.

#### Mixed Domains

A HIA on the Superblocks were projected a mortality reduction of 51 deaths/100,000 deaths (95% UI 18–84) (Table 2) [42]. This HIA recommended combining urban greening with measures discouraging private motorized vehicle us.

Five studies evaluated implemented 20MNs in two Australian cities. In one city, 20MN residents were associated with a significantly higher likelihood of walking for transport (OR 4.24, 95% CI 2.38–7.56) and walked twice as many weekly minutes for transport (82.5 min/week (95% CI 65.3–99.7) versus 41.2 min/week (95% CI 32.7–49.7)) [45]. However, no significant differences were observed in recreational walking or exercise frequency [45, 47], fast-food outlet visits [46], BMI [44], diet [48], or self-rated health [47] (Table 3). For LNG, every 10% increase in compliance was associated with a significantly more transport walking [50] and significantly less psychological distress [51]. Clusters with better community design were associated with significantly more transport walking, total walking, walking > 150 min/week [49], a stronger sense of community, and lower psychological distress [51]. Clusters with better public parkland, movement networks, and lot layout were associated with significantly more recreational walking [49, 50], although mental health outcomes remained unchanged [51].

LNG studies emphasized the need for flexible yet enforceable planning policies [50], clearly defined responsibilities between government and developers [49], and combinations of design features rather than single interventions to promote walking [49]. Reported unplanned effects included spillover of design policies into control areas [50, 51].

#### Differential Effects per Socioeconomic Group

Most studies did not assess whether projected effects differed across socioeconomic groups, and none examined differences by race, ethnicity, or migration background. However, several HIAs suggested that UGS interventions may yield larger projected health benefits in more deprived or socially vulnerable populations. In Bradford, the largest projected mortality reductions occurred in the most deprived quintile (6 deaths/100,000) and in the quintile with the highest proportion of nonwhite residents (11 deaths/100,000), with benefits decreasing across less deprived and ethnically whiter quintiles [32]. Another study reported that all deprivation quintiles presented greater mortality risks compared with the least deprived quintile when lacking green space exposure [29]. In Vienna, higher exposure-attributable mortality rates were projected in lower socioeconomic status (SES) subdistricts [31]. Another study estimated projected mortality differences between the highest and lowest social vulnerability quartiles at 22 deaths/100,000 in Denver, but only 1 death/100,000 in Phoenix [39]. In Barcelona, overweight and obesity were most prevalent in the most deprived quintile at baseline, although the largest projected reductions under the WHO standard occurred in moderately deprived areas [36].

Additionally, one applied policy study found no interaction between 20-min neighborhoods and area- or individual-level SES for physical activity, healthy food consumption, or self-rated health [47].

## Discussion

This review synthesized evidence from 27 studies assessing measured associations and projected health impacts of policy standards. Applied policy standards were associated with favorable outcomes in approximately half of the evaluated measures, although effect sizes varied and many findings were null. HIAs projected mortality benefits from theoretical UGS interventions, whereas applied transport, food, and neighborhood design policies were mainly associated with increased physical activity and healthier diets.

### Urban Green Space

HIAs on UGS policy standards projected benefits for mortality, mental health, child behavior, and diabetes. However, these findings should be interpreted with caution because they were based on modeled projections rather than real-world evaluations. Positive outcomes are predetermined by model specification, as ERFs were aligned with anticipated benefits. Whether these projections translate into real-world health gains remains a key question. A methodological consideration concerns the use of ERFs with confidence intervals including the null value. Such ERFs reflect uncertainty rather than absence of an effect, meaning projected benefits may be compatible with beneficial, null, or small adverse effects. This is relevant for studies such as Iungman et al. (2021) [29] and Mueller et al. (2018) [32]. Therefore, uncertainty intervals and sensitivity analyses are essential when interpreting projected policy impacts.

NDVI and %GA were both used to estimate compliance with the WHO standard. Proposed mechanisms linking UGS to lower mortality include increased physical activity and social interaction, stress reduction, improved immune function, and environmental benefits such as reduced air pollution, noise, and urban heat exposure [58, 59]. Interestingly, NDVI projected larger mortality reductions [35], whereas %GA projected larger reductions in childhood overweight and obesity [36]. The authors attributed these differences to the pathways captured by each metric: %GA better reflects accessible parks that promote physical activity [36], whereas NDVI captures broader greenness that may influence restoration, well-being, and environmental exposures [35].

More ambitious policy standards, such as the WHO standard [35], projected greater benefits but are more challenging to implement. Comparisons across cities were complicated by differing baseline conditions, and many UGS plans lacked detail on accessibility, quality, size, use, and type [29, 31, 32, 35, 37]. Exceptions included the Eixos Verds studies [37, 38], and in the mixed-domains the Superblocks [42] and LNG frameworks [49–51]. Future research should evaluate implemented UGS policies, as existing evidence largely focuses on environmental rather than health outcomes. For example, Berlin’s 500 m and Singapore’s 10-min UGS access policy standards [60, 61] have yet to be evaluated for their health impacts. Overall, UGS policy standards show promise, but their real-world effectiveness and unintended consequences require empirical evaluation.

### Food Environment

Two of the three food stocking studies reported increased healthy food sales, particularly fresh produce and WIC-eligible items, while generating spillover benefits for nearby stores and participating retailers [52, 54]. However, one study found no improvements in dietary quality or caloric purchases [53], highlighting the context-specific nature of these interventions. Food environment studies emphasized the need for complementary strategies to promote healthy eating [52–54] and enforcement for effective implementation [53]. In the mixed domains, the 20MNs showed no significant differences in fast-food outlet visits, diet, or BMI compared to non-20MN neighborhoods [44, 46, 48].

Evidence primarily focused on food stocking policies in American retail settings and food vendor accessibility in the 20MN framework. Although we aimed to include a broader range of food policy standards, interventions such as zoning and restrictions of unhealthy food outlets remain largely unevaluated. For example, the health impacts of recent fast-food bans around schools in England [62] and Mexico [63] have yet to be evaluated. Overall, food environment policy standards may benefit certain communities, but their effectiveness across urban contexts requires further study.

### Transport and Mixed Domain Environment

Studies in the transport and mixed-domain categories demonstrated varying health outcomes shaped by policy design, implementation, and context. HIAs projected reductions in cardiovascular disease (TSMPGT) [43] and mortality (Superblocks) [42]. Applied policies were associated with more transport walking (LNG, LEED_ND, 20MN) [45, 49, 50, 55] and sense of community and mental health outcomes (LNG) [51]. In 20MN studies, no significant differences were found in recreational walking, exercise, food-related outcomes, or BMI compared to non-20MNs [44, 46–48].

Residential self-selection is a mechanism potentially linking underlying lifestyle and neighborhood preferences to both residential location and health-related outcomes. To strengthen causal inference, LNG, LEED_ND, and most 20MN studies adjusted for residential self-selection [45–47, 49–51, 55]. Two 20MN studies did not adjust for this factor [44, 48], but neither study reported statistically significant associations with BMI or dietary outcomes, suggesting that residual residential self-selection was unlikely to have substantially influenced their findings.

A common principle across LNG, 20MN, Superblocks, and LEED_ND was a maximum 400 m (¼ mile) distance to public transport, reflecting a shared approach to healthy urban design. While Superblocks and 20MN are intended broader application, LNG and transport domain policies were more context-specific. One LNG study concluded that improvements in walking result from combinations of urban design elements rather than individual standards, limiting the transferability of isolated policy components [49]. Interestingly, policy spillover occurred as LNG design principles were adopted in conventional developments [49–51]. However, findings from LNG studies should be interpreted cautiously because none of the evaluated developments had been fully completed [50, 51]. Overall, the mixed findings suggest that the effectiveness of built environment standards depends on local context, implementation, and integration with complementary policies.

### Unintended Consequences

Implementing policy standards requires considerations of potential unintended effects. HIAs suggest that greening interventions may reduce socioeconomic health inequalities, although the magnitude and distribution of benefits are context dependent. At the same time, neighborhood improvements may contribute to displacement through green gentrification [64], particularly when affordability is not addressed [30, 38, 65]. Quantitative standards alone, such as proximity thresholds or area-based UGS targets, may overlook quality, resident needs, and capacity, thereby limiting health benefits [6, 66]. Minimum standards may also become default ceilings, particularly in market-driven frameworks such as the applied policy standard LEED_ND, where developers may avoid exceeding minimum requirements to reduce costs [67]. Moreover, health-related financial benefits often accrue to different stakeholders and may take years to materialize [68]. City-level standards can also produce uneven neighborhood effects depending on local context; for example, a new park may improve health in one area while raising safety concerns in another [69]. Although standards can promote equality by ensuring a minimum level of amenities, local adaptation remains essential. Finally, large-scale urban changes may encounter public resistance if implemented through top-down approaches. Participatory, multisectoral approaches, and clear and appealing communication are key to building trust, incorporating local expertise, and legitimizing policies [66, 70, 71].

### Certainty of Evidence

The certainty assessment indicated that confidence in the evidence base was generally low to very low (see Supplementary Table 6). Mortality outcomes were supported by multiple HIAs using established ERFs, but the modeled nature of HIAs introduced important indirectness and uncertainty regarding real-world implementation. Applied policy studies provided more direct evidence than HIAs, but certainty remained limited due to non-randomized designs, heterogeneous findings, imprecise policy definitions, and residual confounding, including residential self-selection.

### Strengths and Limitations

This review has several strengths. This review is the first to systematically assess the health associations and projected effects of built environment policy standards. Moreover, it includes both projected (e.g., HIA) and applied policy standards, offering insights into potential as well as real-world effects to inform policymaking. Additionally, the substantial number of studies screened ensured broad coverage of policy standards across the literature.

Several limitations should be considered when interpreting this systematic review. First, identifying relevant studies proved challenging because of the diverse terminology used for policy standards. We cannot rule out that studies were missed. Second, although we aimed to identify implementation factors and legal frameworks influencing policy effectiveness, these aspects were rarely reported because most included studies focused on impact rather than process evaluation. Third, the wide range of outcomes limited comparability across studies. Fourth, several UGS HIAs were conducted by the same research group via similar methodologies [29–42] and, in four cases, the same ERFs and population data [29, 34, 41, 42]. Consequently, the consistency of findings across UGS HIAs may partly reflect methodological homogeneity, limiting generalizability to other settings. Fifth, most evaluations of implemented policy standards relied on cross-sectional designs, limiting their ability to infer causal effects and underscoring the need for more longitudinal evaluations incorporating before-and-after comparisons. Sixth, although the strength of HIAs lies in their use of causal ERFs from epidemiological evidence, they are limited by their assumptions. This includes full and immediate implementation, immediate health effects, stable population composition, independent risk factors, and often linear exposure-response relationships. Lastly, risk-of-bias assessment was challenging because no validated risk-of-bias tool exists for HIAs. In addition, HIA outcome estimates are subject to uncertainty because they rely on ERFs derived from studies with their own biases and assumptions. Although ROBINS-I was supplemented with elements of PROBAST, it did not adequately distinguish well between higher- and lower-quality HIAs. Therefore, an additional quality appraisal for HIAs was necessary.

## Conclusion

This systematic review highlights both the promise and complexity of policy standards. Many applied policy standards were associated with beneficial health behaviors. HIAs projected health outcomes to various extents. Findings from both study designs are reasons to believe in the potential capacity of policy standards to improve health outcomes, particularly when designed and implemented with attention to equity, quality, and local context, although the overall certainty of evidence was low. Policymakers are increasingly interested in using policy standards to shape healthier built environments, underscoring the need for further research on their real-world health impacts, unintended consequences, and potential to reduce health inequalities. Due to the wide variation among policy standards, further evaluations are necessary to better understand their effects on health.

## Supplementary Information

Below is the link to the electronic supplementary material.

ESM1 (DOCX 376 KB)

ESM2 (DOCX 30.3 KB)

ESM3 (DOCX 19.3 KB)

ESM4 (DOCX 17.2 KB)

ESM5 (DOCX 16.9 KB)

ESM6 (DOCX 16.5 KB)

ESM7 (DOCX 22.4 KB)
