# Supplementary material for: Policy Standards for Healthy Built Environments: A Systematic Review of Health Impact Assessments and Nonrandomized Evaluations of Implemented Policies
Source: J Urban Health. 2026 Sep 19;103(5):908–48. doi: 10.1007/s11524-026-01137-4 (PMC13672719; doi:10.1007/s11524-026-01137-4)
Supplement: Supplementary file 3 [file 11524_2026_1137_MOESM3_ESM.docx]

**Supplementary Table 4. Search strategy.**

| **Database searched** | **Platform** | **Years of coverage** | **Records** | **Records after duplicates removed** |
| --- | --- | --- | --- | --- |
| Medline ALL | Ovid | 1946 - Present | 3941 | 3904 |
| Embase | Embase.com | 1971 - Present | 5462 | 2562 |
| Web of Science Core Collection* | Web of Knowledge | 1975 - Present | 5727 | 3489 |
| PsycINFO | Ovid | 1806 - Present | 1287 | 636 |
| Cochrane Central Register of Controlled Trials** | Wiley | 1992 - Present | 48 | 6 |
| International Bibliography of Social Sciences (IBSS) | ProQuest | 1951-present | 393 | 165 |
| Social Science Database‎ | ProQuest |  | 221 | 60 |
| Applied Social Sciences Index & Abstracts (ASSIA)‎ | ProQuest | 1987-present | 105 | 14 |
| Social Services Abstracts‎ | ProQuest | 1979-present | 61 | 14 |
| Sociological Abstracts‎ | ProQuest | 1952-present | 197 | 84 |
| Sociology Database‎ | ProQuest | 1985-present | 59 | 2 |
| **Total** | | | **17501** | **10936** |

*Science Citation Index Expanded (1975-present); Social Sciences Citation Index (1975-present) ; Arts & Humanities Citation Index (1975-present) ; Conference Proceedings Citation Index- Science (1990-present) ; Conference Proceedings Citation Index- Social Science & Humanities (1990-present) ; Emerging Sources Citation Index (2005-present)

*Exact search turned on in Web of Science Core Collection

** Manually deleted abstracts from trial registries

**Medline**

(Policy / OR Public Policy / OR City Planning / OR Program Evaluation / OR Environmental Policy / OR (policy OR policies OR ((city OR urban OR design*) ADJ3 (plan OR planning OR norm* OR standard* OR recommendat* OR guideline* OR rule* OR implement*)) OR min-neighbourhood* OR min-city* OR min-cities* OR min-neighborhood* OR minute-neighbourhood* OR minute-city* OR minute-cities* OR compact-cit* OR minute-neighborhood* OR 3-30-300-rule* OR goal* OR (program* ADJ3 evaluat*) OR leverag* OR authori* OR code OR codes OR regulation*).ab,ti,kw.) AND (* City Planning / OR * Environmental Policy / OR * Built Environment / OR * Social Environment / OR * Parks, Recreational / OR (((city OR cities OR urban OR environment* OR neighbourhood* OR neighborhood* OR transport* OR suburb*) ADJ3 (plan OR planning OR norm* OR standard* OR recommendat* OR guideline* OR rule* OR design* OR walkable OR walkfriend* OR walk-friend* OR bikeable OR bikefriend* OR bike-friend* OR change*)) OR greenway* OR alleyway* OR ((living OR built OR food OR social OR traffic OR transport OR green OR open OR public OR activity OR obesity OR obesogenic OR neighbourhood* OR neighborhood* OR street* OR physical*) ADJ3 (environment*)) OR foodscape OR (healthy-food* ADJ3 (accessib* OR availab*)) OR ((green OR open OR public OR activity) ADJ3 (area OR space)) OR greenspace* OR bikeability OR walkability OR min-neighbourhood* OR min-city* OR min-cities* OR min-neighborhood* OR minute-neighbourhood* OR minute-city* OR minute-cities* OR compact-cit* OR minute-neighborhood* OR 3-30-300-rule* OR (tree ADJ3 cover ADJ3 goal*) OR (cycling ADJ3 network*) OR bike-lane* OR bikelane*).ti.) AND (Quality of Life/ OR exp Diet/ OR Eating / OR exp Food Insecurity / OR Food Security / OR Food Deserts / OR Exercise/ OR Sedentary Behavior / OR Health Behavior/ OR exp Smoking/ OR Life Style / OR exp Mortality/ OR Leisure Activities/ OR Stress, Psychological / OR Social Interaction/ OR Body Weight/ OR Body Mass Index/ OR Obesity/ OR Health Impact Assessment/ OR * Public Health/ OR Healthcare Disparities / OR Health Status Disparities / OR Socioeconomic Disparities in Health / OR Health Inequities / OR (HRQOL OR QOL OR BMI OR diet OR nutrition OR dietary OR lifestyle OR life-style OR mortalit* OR leisure OR bodyweight* OR body-weight* OR overweight* OR body-mass OR obesity OR sleep* OR ((health) ADJ3 (impact)) OR exercise* OR cycling OR walking OR smoking OR stress* OR relax* OR ((physical*) ADJ (activ* OR inactiv*)) OR ((quality) ADJ3 (life)) OR ((food OR alcohol OR fat OR vitamin*) ADJ3 (intake* OR sales OR consumption* OR access* OR securit* OR insecurit* OR desert*)) OR sedentary OR ((eating OR drinking OR social*) ADJ3 (behavior OR behaviour)) OR ((social*) ADJ3 (interaction* OR functioning)) OR (health ADJ3 (disparit* OR equit* OR inequit* OR equal* OR inequal*))).ab,ti,kw. OR (public-health OR healthy OR healthier).ti.) NOT ((animal/ OR animal*:de OR nonhuman/) NOT (human/)) NOT (news OR congres* OR abstract* OR book* OR chapter* OR dissertation abstract*).pt.

**Embase**

('policy'/de OR 'public policy'/exp OR 'city planning'/de OR 'program evaluation'/de OR 'environmental planning'/de OR (policy OR policies OR ((city OR urban OR design*) NEAR/3 (plan OR planning OR norm* OR standard* OR recommendat* OR guideline* OR rule* OR implement*)) OR min-neighbourhood* OR min-city* OR min-cities* OR min-neighborhood* OR minute-neighbourhood* OR minute-city* OR minute-cities* OR compact-cit* OR minute-neighborhood* OR 3-30-300-rule* OR goal* OR (program* NEAR/3 evaluat*) OR leverag* OR authori* OR code OR codes OR regulation*):ab,ti,kw) AND ('city planning'/mj/de OR 'environmental planning'/mj/exp OR 'obesogenic environment'/mj/de OR 'built environment'/mj/de OR 'food environment'/mj/de OR 'social environment'/mj/de OR 'green space'/mj/de OR (((city OR cities OR urban OR environment* OR neighbourhood* OR neighborhood* OR transport* OR suburb*) NEAR/3 (plan OR planning OR norm* OR standard* OR recommendat* OR guideline* OR rule* OR design* OR walkable OR walkfriend* OR walk-friend* OR bikeable OR bikefriend* OR bike-friend* OR change*)) OR greenway* OR alleyway* OR ((living OR built OR food OR social OR traffic OR transport OR green OR open OR public OR activity OR obesity OR obesogenic OR neighbourhood* OR neighborhood* OR street* OR physical*) NEAR/3 (environment*)) OR foodscape OR (healthy-food* NEAR/3 (accessib* OR availab*)) OR ((green OR open OR public OR activity) NEAR/3 (area OR space)) OR greenspace* OR bikeability OR walkability OR min-neighbourhood* OR min-neighborhood* OR minute-neighbourhood* OR compact-cit* OR minute-neighborhood* OR 3-30-300-rule* OR (tree NEAR/3 cover NEAR/3 goal*) OR (cycling NEAR/3 network*) OR bike-lane* OR bikelane*):ti) AND ('quality of life'/exp OR 'diet'/de OR 'nutrition'/de OR 'dietary intake'/exp OR 'food access'/exp OR 'exercise'/exp OR 'physical activity'/exp OR 'physical inactivity'/exp OR 'health behavior'/exp OR 'smoking'/exp OR 'lifestyle'/exp OR Mortality/exp OR 'leisure'/de OR 'mental stress'/exp OR 'social interaction'/de OR 'body weight'/exp OR 'body mass'/de OR 'obesity'/de OR 'obesity'/exp OR 'health impact assessment'/de OR 'public health'/mj OR 'health disparity'/de OR (HRQOL OR QOL OR BMI OR diet OR nutrition OR dietary OR lifestyle OR life-style OR mortalit* OR leisure OR bodyweight* OR body-weight* OR overweight* OR body-mass OR obesity OR sleep* OR ((health) NEAR/3 (impact)) OR exercise* OR cycling OR walking OR smoking OR stress* OR relax* OR ((physical*) NEXT/1 (activ* OR inactiv*)) OR ((quality) NEAR/3 (life)) OR ((food OR alcohol OR fat OR vitamin*) NEAR/3 (intake* OR sales OR consumption* OR access* OR securit* OR insecurit* OR desert*)) OR sedentary OR ((eating OR drinking OR social*) NEAR/3 (behavior OR behaviour)) OR ((social*) NEAR/3 (interaction* OR functioning)) OR (health NEAR/3 (disparit* OR equit* OR inequit* OR equal* OR inequal*))):ab,ti,kw OR (public-health OR healthy OR healthier):ti) NOT ((animal/exp OR animal*:de OR nonhuman/de) NOT ('human'/exp)) NOT ([conference abstract]/lim)

**Web of science**

TS=((policy OR policies OR ((city OR urban OR design*) NEAR/2 (plan OR planning OR norm* OR standard* OR recommendat* OR guideline* OR rule* OR implement*)) OR min-neighbourhood* OR min-city* OR min-cities* OR min-neighborhood* OR minute-neighbourhood* OR minute-city* OR minute-cities* OR compact-cit* OR minute-neighborhood* OR 3-30-300-rule* OR goal* OR (program* NEAR/2 evaluat*) OR leverag* OR authori* OR code OR codes OR regulation*)) AND TI=((((city OR cities OR urban OR neighbourhood* OR neighborhood* OR transport* OR suburb*) NEAR/2 (plan OR planning OR norm* OR standard* OR recommendat* OR guideline* OR rule* OR design* OR walkable OR walkfriend* OR walk-friend* OR bikeable OR bikefriend* OR bike-friend* OR change*)) OR greenway* OR alleyway* OR ((living OR built OR food OR social OR traffic OR transport OR green OR open OR public OR activity OR obesity OR obesogenic OR neighbourhood* OR neighborhood* OR street* OR physical*) NEAR/2 (environment*)) OR foodscape OR (healthy-food* NEAR/2 (accessib* OR availab*)) OR ((green OR open OR public OR activity) NEAR/2 (area OR space)) OR greenspace* OR bikeability OR walkability OR min-neighbourhood* OR min-neighborhood* OR minute-neighbourhood* OR compact-cit* OR minute-neighborhood* OR 3-30-300-rule* OR (tree NEAR/2 cover NEAR/2 goal*) OR (cycling NEAR/2 network*) OR bike-lane* OR bikelane*)) AND (TS=(HRQOL OR QOL OR BMI OR diet OR nutrition OR dietary OR lifestyle OR life-style OR mortalit* OR leisure OR bodyweight* OR body-weight* OR overweight* OR body-mass OR obesity OR sleep* OR ((health) NEAR/2 (impact)) OR exercise* OR cycling OR walking OR smoking OR stress* OR relax* OR ((physical*) NEAR/1 (activ* OR inactiv*)) OR ((quality) NEAR/2 (life)) OR ((food OR alcohol OR fat OR vitamin*) NEAR/2 (intake* OR sales OR consumption* OR access* OR securit* OR insecurit* OR desert*)) OR sedentary OR ((eating OR drinking OR social*) NEAR/2 (behavior OR behaviour)) OR ((social*) NEAR/2 (interaction* OR functioning)) OR (health NEAR/2 (disparit* OR equit* OR inequit* OR equal* OR inequal*))) OR TI=(public-health OR healthy OR healthier)) NOT DT=(Meeting Abstract OR Meeting Summary)

**Cochrane**

((policy OR policies OR ((city OR urban OR design*) NEAR/3 (plan OR planning OR norm* OR standard* OR recommendat* OR guideline* OR rule* OR implement*)) OR min-neighbourhood* OR min-city* OR min-cities* OR min-neighborhood* OR minute-neighbourhood* OR minute-city* OR minute-cities* OR compact-cit* OR minute-neighborhood* OR "3-30-300-rule" OR goal* OR (program* NEAR/3 evaluat*) OR leverag* OR authori* OR code OR codes OR regulation*):ab,ti)

((((city OR cities OR urban OR environment* OR neighbourhood* OR neighborhood* OR transport* OR suburb*) NEAR/3 (plan OR planning OR norm* OR standard* OR recommendat* OR guideline* OR rule* OR design* OR walkable OR walkfriend* OR walk-friend* OR bikeable OR bikefriend* OR bike-friend* OR change*)) OR greenway* OR alleyway* OR ((living OR built OR food OR social OR traffic OR transport OR green OR open OR public OR activity OR obesity OR obesogenic OR neighbourhood* OR neighborhood* OR street* OR physical*) NEAR/3 (environment*)) OR foodscape OR (healthy-food* NEAR/3 (accessib* OR availab*)) OR ((green OR open OR public OR activity) NEAR/3 (area OR space)) OR greenspace* OR bikeability OR walkability OR min-neighbourhood* OR min-neighborhood* OR minute-neighbourhood* OR compact-cit* OR minute-neighborhood* OR "3-30-300-rule" OR (tree NEAR/3 cover NEAR/3 goal*) OR (cycling NEAR/3 network*) OR bike-lane* OR bikelane*):ti)

((HRQOL OR QOL OR BMI OR diet OR nutrition OR dietary OR lifestyle OR life-style OR mortalit* OR leisure OR bodyweight* OR body-weight* OR overweight* OR body-mass OR obesity OR sleep* OR ((health) NEAR/3 (impact)) OR exercise* OR cycling OR walking OR smoking OR stress* OR relax* OR ((physical*) NEXT (activ* OR inactiv*)) OR ((quality) NEAR/3 (life)) OR ((food OR alcohol OR fat OR vitamin*) NEAR/3 (intake* OR sales OR consumption* OR access* OR securit* OR insecurit* OR desert*)) OR sedentary OR ((eating OR drinking OR social*) NEAR/3 (behavior OR behaviour)) OR ((social*) NEAR/3 (interaction* OR functioning)) OR (health NEAR/3 (disparit* OR equit* OR inequit* OR equal* OR inequal*))):ab,ti OR (public-health OR healthy OR healthier):ti)

("conference abstract":kw OR Trial registry record:pt)

#1 AND #2 AND #3 NOT #4

**PsycINFO**

(exp Policy Making / OR Urban Planning / OR Program Evaluation / OR Environmental Policy / OR (policy OR policies OR ((city OR urban OR design*) ADJ3 (plan OR planning OR norm* OR standard* OR recommendat* OR guideline* OR rule* OR implement*)) OR min-neighbourhood* OR min-city* OR min-cities* OR min-neighborhood* OR minute-neighbourhood* OR minute-city* OR minute-cities* OR compact-cit* OR minute-neighborhood* OR 3-30-300-rule* OR goal* OR (program* ADJ3 evaluat*) OR leverag* OR authori* OR code OR codes OR regulation*).ab,ti.) AND (* Urban Planning / OR * Environmental Policy / OR * Built Environment / OR * Social Environments/ OR * Recreation Areas / OR (((city OR cities OR urban OR environment* OR neighbourhood* OR neighborhood* OR transport* OR suburb*) ADJ3 (plan OR planning OR norm* OR standard* OR recommendat* OR guideline* OR rule* OR design* OR walkable OR walkfriend* OR walk-friend* OR bikeable OR bikefriend* OR bike-friend* OR change*)) OR greenway* OR alleyway* OR ((living OR built OR food OR social OR traffic OR transport OR green OR open OR public OR activity OR obesity OR obesogenic OR neighbourhood* OR neighborhood* OR street* OR physical*) ADJ3 (environment*)) OR foodscape OR (healthy-food* ADJ3 (accessib* OR availab*)) OR ((green OR open OR public OR activity) ADJ3 (area OR space)) OR greenspace* OR bikeability OR walkability OR min-neighbourhood* OR min-neighborhood* OR minute-neighbourhood* OR compact-cit* OR minute-neighborhood* OR 3-30-300-rule* OR (tree ADJ3 cover ADJ3 goal*) OR (cycling ADJ3 network*) OR bike-lane* OR bikelane*).ti.) AND ("Quality of Life"/ OR exp Diets/ OR Eating Behavior/ OR exp Food Insecurity / OR Exercise/ OR Sedentary Behavior / OR Health Behavior/ OR exp Tobacco Smoking / OR Smoking Cessation / OR Lifestyle / OR Leisure Time/ OR Psychological Stress / OR Social Interaction/ OR Body Weight/ OR Body Mass Index/ OR Obesity/ OR * Public Health/ OR Health Disparities / OR (HRQOL OR QOL OR BMI OR diet OR nutrition OR dietary OR lifestyle OR life-style OR mortalit* OR leisure OR bodyweight* OR body-weight* OR overweight* OR body-mass OR obesity OR sleep* OR ((health) ADJ3 (impact)) OR exercise* OR cycling OR walking OR smoking OR stress* OR relax* OR ((physical*) ADJ (activ* OR inactiv*)) OR ((quality) ADJ3 (life)) OR ((food OR alcohol OR fat OR vitamin*) ADJ3 (intake* OR sales OR consumption* OR access* OR securit* OR insecurit* OR desert*)) OR sedentary OR ((eating OR drinking OR social*) ADJ3 (behavior OR behaviour)) OR ((social*) ADJ3 (interaction* OR functioning)) OR (health ADJ3 (disparit* OR equit* OR inequit* OR equal* OR inequal*))).ab,ti. OR (public-health OR healthy OR healthier).ti.) NOT ((animal.po. OR exp animals/) NOT human.po.) NOT (news OR congres* OR abstract* OR book* OR chapter* OR dissertation abstract*).pt.

**International Bibliography of the Social Sciences (IBSS)‎**

**Social Science Database‎**

**Applied Social Sciences Index & Abstracts (ASSIA)‎**

**Social Services Abstracts‎**

**Sociological Abstracts‎**

**Sociology Database‎**

AB,TI((policy OR policies OR ((city OR urban OR design*) N/3 (plan OR planning OR norm* OR standard* OR recommendat* OR guideline* OR rule* OR implement*)))) AND TI((((city OR cities OR urban OR environment* OR neighbourhood* OR neighborhood* OR transport* OR suburb*) N/3 (plan OR planning OR norm* OR standard* OR recommendat* OR guideline* OR rule* OR design* OR walkable OR walkfriend* OR walk-friend* OR bikeable OR bikefriend* OR bike-friend* OR change*)) OR greenway* OR alleyway* OR (healthy-food* N/3 (accessib* OR availab*)) OR ((green OR open OR public OR activity) N/3 (area OR space)) OR greenspace* OR bikeability OR walkability OR bike-lane* OR bikelane*)) AND (AB,TI(diet OR nutrition OR dietary OR lifestyle OR life-style OR mortalit* OR leisure OR bodyweight* OR body-weight* OR overweight* OR body-mass OR obesity OR sleep* OR ((health) N/3 (impact)) OR exercise* OR smoking OR stress* OR relax* OR ((physical*) N/1 (activ* OR inactiv*)) OR ((quality) N/3 (life)) OR sedentary OR ((eating OR drinking OR social*) N/3 (behavior OR behaviour)) OR (health N/3 (disparit* OR equit* OR inequit* OR equal* OR inequal*))))
