# Supplementary material for: Policy Standards for Healthy Built Environments: A Systematic Review of Health Impact Assessments and Nonrandomized Evaluations of Implemented Policies
Source: J Urban Health. 2026 Sep 19;103(5):908–48. doi: 10.1007/s11524-026-01137-4 (PMC13672719; doi:10.1007/s11524-026-01137-4)
Supplement: Supplementary file 4 [file 11524_2026_1137_MOESM4_ESM.docx]

**Supplementary Table 5. Quality appraisal of included HIAs.**

| **Study** | **Quality and appropriateness of ERFs (0-3)** | **Plausibility of exposure scenario (0-3)** | **Treatment of uncertainty/sensitivity (0-2)** | **Transferability of ERFs (demographic, socioeconomic and environmental setting) (0-3)** | **Transparency and reproducibility (0-2)** | **Total** |
| --- | --- | --- | --- | --- | --- | --- |
|  | 3: Recent meta-analysis  2: High-quality systematic review or large cohort study 1: Single study with limitations or indirect evidence 0: ERF source unclear or unsupported | 3: Detailed implementation plans  2: Reasonably justified but with assumptions insufficiently supported 1: Based on speculative assumptions 0: Unrealistic or poorly described | 2: Comprehensive sensitivity analyses conducted and interpreted 1: Limited sensitivity analysis conducted 0: No sensitivity analysis | 3: Population and context closely match derivation studies 2: Mostly comparable with minor differences 1: Important contextual differences not addressed 0: ERF applied to clearly dissimilar population/context | 2: Methods and assumptions clearly reproducible  1: Partial transparency  0: Insufficient detail | **0-13** |
| Mueller (2017 -1) | 3 | 2 | 0 | 2 | 2 | 9 |
| Mueller (2017-2) | 1 | 2 | 0 | 3 | 2 | 8 |
| Iungman (2021) | 3 | 2 | 2 | 2 | 2 | 11 |
| Mueller (2018) | 3 | 2 | 0 | 2 | 2 | 9 |
| Mueller (2020) | 3 | 3 | 0 | 2 | 2 | 10 |
| Khomenko (2020) | 3 | 2 | 2 | 2 | 2 | 11 |
| Kondo (2020) | 3 | 2 | 2 | 2 | 2 | 11 |
| Wang (2022) | 1 | 3 | 0 | 0 | 0 | 4 |
| Barboza (2021) | 3 | 2 | 2 | 2 | 2 | 11 |
| Vidal-Yañez (2023) | 1 | 3 | 2 | 3 | 2 | 11 |
| Opbroek (2024) | 1 | 3 | 2 | 3 | 2 | 11 |
| Iungman (2025) | 3 | 3 | 1 | 3 | 2 | 12 |
| Iungman (2023) | 3 | 2 | 2 | 3 | 2 | 12 |
| Nguyen (2024) | 3 | 2 | 1 | 2 | 2 | 10 |
| Dean  (2024) | 3 | 2 | 2 | 3 | 2 | 12 |
