# Supplementary material for: Policy Standards for Healthy Built Environments: A Systematic Review of Health Impact Assessments and Nonrandomized Evaluations of Implemented Policies
Source: J Urban Health. 2026 Sep 19;103(5):908–48. doi: 10.1007/s11524-026-01137-4 (PMC13672719; doi:10.1007/s11524-026-01137-4)
Supplement: Supplementary file 5 [file 11524_2026_1137_MOESM5_ESM.docx]

| **Supplementary Table 6. Certainty of evidence assessment.** | | | | | | | |  |
| --- | --- | --- | --- | --- | --- | --- | --- | --- |
| **Outcome** | **N studies** | **Study design** | **Risk of bias / quality score** | **Inconsistency** | **Indirectness** | **Imprecision** | **Other considerations** | **Certainty** |
| All-cause/natural-cause mortality | 9 | HIA | 9-12 | NA | Serious | Serious | ERFs from meta-analyses | LOW |
| Heat-related mortality | 2 | HIA | 12 | NA | Serious | Serious | ERF from meta-analysis, but cooling effects are modeled | LOW |
| Cardiovascular morbidity | 1 | HIA | 4 | NA | Serious | Very serious | ERF from meta-analysis, but intermediate exposure-PA step is modeled | VERY LOW |
| Stroke/dementia | 1 | HIA | 12 | NA | Serious | Not serious | ERF from large cohort study | LOW |
| Childhood overweight/obesity | 1 | HIA | 10 | NA | Serious | Not Serious | Single HIA; exposure assumptions uncertain | VERY LOW |
| Mental health outcomes | 2 | HIA | 8-11 | NA | Serious | Serious | ERF from cross-sectional study from local data | VERY LOW |
| Child cognitive development | 1 | HIA | 11 | NA | Serious | Serious | ERF from cross-sectional study from local data | VERY LOW |
| Walking/physical activity | 4 | Quasi-experimental, cross-sectional observational | Moderate | Serious | Not serious | Serious | Several studies; all corrected for residential self- selection. Some with non-precise policy | LOW |
| Children’s MVPA | 1 | Cross-sectional observational | Moderate | NA | Not serious | Serious | Single study with small sample size. Corrected for residential self-selection | LOW |
| Sense of community/ psychological distress | 1 | Quasi-experimental | Moderate | NA | Not serious | Serious | Non-precise policy, corrected for residential self-selection | LOW |
| Food consumption | 6 | Quasi-experimental, cross-sectional observational, case study | Serious | Serious | Not serious | Serious | Findings mixed, some findings without analytical method | LOW |
| BMI/obesity | 1 | Cross-sectional observational | Moderate | NA | Not serious | Serious | Residential self-selection is a concern | LOW |
| Self-rated health | 1 | Cross-sectional observational | Moderate | NA | Not serious | Serious | Null findings | LOW |
