# Supplementary material for: Policy Standards for Healthy Built Environments: A Systematic Review of Health Impact Assessments and Nonrandomized Evaluations of Implemented Policies
Source: J Urban Health. 2026 Sep 19;103(5):908–48. doi: 10.1007/s11524-026-01137-4 (PMC13672719; doi:10.1007/s11524-026-01137-4)
Supplement: Supplementary file 6 [file 11524_2026_1137_MOESM6_ESM.docx]

**Supplementary Table 7: Risk of bias assessment overview based on ROBINS-I and PROBAST (for selection bias)**

| **Study** | **Bias due to confounding** | **Bias in selection of participants** | **Bias in classification of interventions** | **Bias due to deviations from interventions** | **Bias due to missing data** | **Bias in measurement of outcomes** | **Bias in selection of the reported result** | **Overall Bias** |
| --- | --- | --- | --- | --- | --- | --- | --- | --- |
| **Hooper (2014)** | Moderate | Low | Low | Moderate | Low | Low | Low | Moderate |
| **Hooper (2015)** | Moderate | Low | Low | Moderate | Low | Moderate | Low | Moderate |
| **Contardo Ayala (2022)** | Moderate | Low | Low | Low | Low | Moderate | Low | Moderate |
| **Minkler (2018** | Moderate | Moderate | Low | Moderate | Serious | Low | Low | Serious |
| **Laska (2019)** | Moderate | Low | Low | Low | Low | Low | Low | Moderate |
| **Hudak (2020)** | Moderate | Moderate | Low | Low | Moderate | Low | Low | Moderate |
| **Hooper (2018)** | Moderate | Low | Low | Moderate | Low | Low | Low | Moderate |
| **Yang (2022)** | Moderate | Low | Low | Low | Low | Moderate | Low | Moderate |
| **Stevens (2011)** | Moderate | Low | Low | Low | Moderate | Moderate | Low | Moderate |
| **Oostenbach (2022)** | Moderate | Low | Low | Low | Low | Low | Low | Moderate |
| **Oostenbach (2023)** | Moderate | Low | Low | Low | Moderate | Low | Low | Moderate |
| **Lamb (2023)** | Moderate | Low | Low | Low | Moderate | Low | Low | Moderate |
