# Supplementary material for: Policy Standards for Healthy Built Environments: A Systematic Review of Health Impact Assessments and Nonrandomized Evaluations of Implemented Policies
Source: J Urban Health. 2026 Sep 19;103(5):908–48. doi: 10.1007/s11524-026-01137-4 (PMC13672719; doi:10.1007/s11524-026-01137-4)
Supplement: Supplementary file 7 [file 11524_2026_1137_MOESM7_ESM.docx]

**Supplementary Table 8. Calculations for comparable results.**

| **Author** | **Standard** | **Calculations per 100,000 population** |
| --- | --- | --- |
| Mueller (2017-1) [34] | **WHO universal access to UGS** (≥0.5 ha green space within 300 m from residence) | Barcelona: 116 annual deaths prevented on a population of n=1,357,361.  Current mortality rate: 1,108 deaths/100,000.  *Deaths/100,000*  *116*(100,000/1,357,361) = 9 deaths/100,000 prevented.*  *New mortality rate = 1,108 - 9 = 1,099 deaths/100,000.*  *The upper and lower bound of the UI were calculated in a similar fashion, but by plugging in the lower and upper bound of the 95%CI. Example upper bound:*  *236 *(100,000/1,357,361) = 17 deaths/100,000 prevented*  *Percentage*  *9 deaths/100,000 / 1,108 deaths/100,000 * 100% = 0.8%*  *The upper and lower bound of the percentage UI were calculated in a similar fashion, but by plugging in the lower and upper bound of the UI. Example upper bound:*  *17 deaths/100,000 / 1,108 deaths/100,000 * 100% = 1.5%* |
| Mueller (2017-2) [33] | **WHO universal access to UGS (**≥0.5 ha green space within 300 m from residence) | Barcelona: 740 cases of depression prevented on a population of n=1,357,361.  Current depression Incidence rate: 895/100,000.  *Cases/100,000*  *740*(100,000/1,357,361) = 55 cases/100,000 prevented.*  *New incidence rate = 895-55=840 cases/100,000.*  *The upper and lower bound of the UI were calculated in a similar fashion, but by plugging in the lower and upper bound of the 95%CI. Example upper bound:*  *1,199 *(100,000/1,357,361) = 88 cases/100,000 prevented.*  *Percentage*  *55 cases/100,000 / 895 cases/100,000 * 100% = 6.0%*  *The upper and lower bound of the percentage UI were calculated in a similar fashion, but by plugging in the lower and upper bound of the UI. Example upper bound:*  *88 cases /100,000 / 895 cases/100,000 * 100% = 9.8%*  *6.0% (2.5%-9.8%)* |
| Mueller (2018) [32] | **WHO universal access to UGS (**≥0.5 ha green space within 300 m from residence) | Bradford: 16 deaths prevented on a population of n=393,091.  Current mortality 1,114 deaths/100,000.  *Deaths/100,000*  *16*(100,000/393,091) = 4 deaths/100,000 prevented.*  *New mortality rate = 1,114 – 4 = 1,110 deaths/100,000.*  *The upper and lower bound of the UI were calculated in a similar fashion, but by plugging in the lower and upper bound of the 95%CI. Example upper bound:*  *32 *(100,000/393,091) = 8 deaths/100,000 prevented.*  *Percentage*  *4 deaths/100,000 / 1,114 deaths/100,000 * 100% = 0.3%*  *The upper and lower bound of the percentage UI were calculated in a similar fashion, but by plugging in the lower and upper bound of the UI. Example upper bound:*  *8 deaths/100,000 / 1,114 deaths/100,000 * 100% = 0.7%* |
| Mueller (2020) [42] | **Superblocks** (grid structured neighborhoods (1x1 blocks) are transformed to 3x3 traffic-calmed superblocks) | Barcelona: 667 deaths prevented on a population of n=1,301,827.  Current mortality rate: 1,144 deaths/100,000.  *Deaths/100,000*  *667*(100,000/1,301,827) =51 deaths/100,000 prevented.*  *New mortality rate = 1093 deaths/100,000.*  *The upper and lower bound of the UI were calculated in a similar fashion, but by plugging in the lower and upper bound of the 95%CI. Example upper bound:*  *1,098*(100,000/1,301,827) = 84 deaths/100,000 prevented.*  *Percentage*  *51 deaths/100,000 / 1,114 deaths/100,000 * 100% = 4.5%*  *The upper and lower bound of the percentage UI were calculated in a similar fashion, but by plugging in the lower and upper bound of the UI. Example upper bound:*  *84 deaths/100,000 / 1,114 deaths/100,000 * 100% = 7.3%.* |
| Khomenko (2020) [31] | **WHO universal access to UGS** (≥0.5 ha green space within 300 m from residence)  **Viennese policy**  (≥3.5 m² UGS/inhabitant)  **Alternative WHO policy standard** (≥9 m² UGS/inhabitant) | Vienna: population n = 1,488,832.  Current mortality rate=15,581*(100,000/1,488,832) =1,047 deaths/100,000.   - *Universal access (WHO): 190 deaths prevented.*   *Deaths/100,000*  *190 * (100,000/1,488,832) = 13 deaths/100,000 prevented.*  *New mortality rate = 1,047 -13 = 1,034 deaths/100,000*  *The upper and lower bound of the UI were calculated in a similar fashion, but by plugging in the lower and upper bound of the 95%CI. Example upper bound universal access:*  *386 * (100,000/1,488,832) = 26 deaths/100,000 prevented.*  *Percentage*  *13 deaths/100,000 / 1,047 deaths/100,000 * 100% = 1.2%*  *The upper and lower bound of the percentage UI were calculated in a similar fashion, but by plugging in the lower and upper bound of the UI. Example upper bound:*  *26 deaths/100,000 / 1,047 deaths/100,000 * 100% = 2.4%.*   - *Viennese policy: 26 deaths prevented.*   *26 * (100,000/1,488,832) = 2 deaths/100,000 prevented.*  *New mortality rate = 1,047 -2 = 1,045 deaths/100,000*   - *Alternative WHO recommendation: 114 deaths prevented.*   *114 * (100,000/1,488,832) = 8 deaths/100,000 prevented.*  *New mortality rate = 1,047 -8 = 1,039 deaths/100,000.*  *Percentages as well as upper and lower bounds were calculated in the same fashion as in the WHO standard.* |
| Iungman (2021) [29] | **WHO universal access to UGS** (≥0.5 ha green space within 300 m from residence) | - Barcelona: 277 deaths prevented on a population of n=1,348,800.   Current mortality rate: 1,082 deaths/100,000.  Deaths/100,000  *277*(100,000/1,348,800) =21 deaths/100,000 prevented.*  *New mortality rate = 1082-20=1062 deaths/100,000*  *The upper and lower bound of the UI for Barcelona and Madrid were calculated in a similar fashion, but by plugging in the lower and upper bound of the 95%CI. Example upper bound (Barcelona):*  *581 *(100,000/1,348,800) = 43 deaths/100,000 prevented*  *Percentage*  *21 deaths/100,000 / 1,082 deaths/100,000 * 100% = 1.9%*  *The upper and lower bound of the percentage UI were calculated in a similar fashion, but by plugging in the lower and upper bound of the UI. Example upper bound:*  *43 deaths/100,000 / 1,114 deaths/100,000 * 100% = 3.9%.*   - Madrid: 337 deaths prevented on a population of n =2,649,734.   Current mortality rate 977 deaths/100,000.  *Deaths/100,000*  *337*(100,000/2,649,734) =13 deaths/100,000 prevented.*  *New mortality rate = 977-14 = 963 deaths/100,000*  *Percentage*  *13 deaths/100,000 / 977 deaths/100,000 * 100% = 1.3%*  *The upper and lower bound of the percentage UI were calculated in a similar fashion, but by plugging in the lower and upper bound of the UI. Example upper bound:*  *26 deaths/100,000 / 977 deaths/100,000 * 100% = 2.6%.* |
| Iungman (2025) [41] | **Eixos Verds** (transforms one-third of the streets into green corridors) | 178 deaths prevented (NDVI); 5 deaths through lower temperature  Current mortality rate: 1,054/100,000  *Calculating the Barcelona population through algebra*  *178 deaths * (100,000/X) = 13 deaths/100,000*  *100,000/X = (13/178)*  *100,000/X = 0.07*  *X = 100,000/0.07*  *X = 1,369,231 = Barcelona population*  *Temperature deaths/100,000*  *5 deaths * (100,000/1,369,231) = 0.4 deaths/100,000*  *The upper and lower bound of the UI were calculated in a similar fashion, but by plugging in the lower and upper bound of the 95%CI. Example upper bound:*  *6 deaths * (100,000/1,369,231) = 0.4 deaths/100,000*  *Temperature percentage*  *0.4 deaths/100,000 / 1,054 deaths/100,000 * 100% = 0.03%*  *The upper and lower bound of the percentage UI were calculated in a similar fashion, but by plugging in the lower and upper bound of the UI. Example upper bound:*  *0.4 deaths/100,000 / 1,054 deaths/100,000 * 100% = 0.03%.* |
