# Supplementary material for: Policy Standards for Healthy Built Environments: A Systematic Review of Health Impact Assessments and Nonrandomized Evaluations of Implemented Policies
Source: J Urban Health. 2026 Sep 19;103(5):908–48. doi: 10.1007/s11524-026-01137-4 (PMC13672719; doi:10.1007/s11524-026-01137-4)
Supplement: Supplementary file 8 [file 11524_2026_1137_MOESM1_ESM.docx]

***Supplementary Fig. 2*** *Geographical distribution of the included studies*
